# Composite genome sequence of *Bacillus clausii*, a probiotic commercially available as Enterogermina^®^, and insights into its probiotic properties

**DOI:** 10.1186/s12866-019-1680-7

**Published:** 2019-12-30

**Authors:** Indu Khatri, Gaurav Sharma, Srikrishna Subramanian

**Affiliations:** 1 CSIR-Institute of Microbial Technology, Sector-39A, Chandigarh, 160036 India; 2 Leiden University Medical Center, Leiden, the Netherlands; 3 Institute of Bioinformatics and Applied Biotechnology, Bengaluru, Karnataka India

**Keywords:** Bacteriocins, Gastrointestinal-tract, Phylogeny, Resistome, Pathogenicity

## Abstract

**Background:**

Some of the spore-forming strains of *Bacillus* probiotics are marketed commercially as they survive harsh gastrointestinal conditions and bestow health benefits to the host.

**Results:**

We report the composite genome of *Bacillus clausii* ENTPro from a commercially available probiotic Enterogermina**®** and compare it with the genomes of other *Bacillus* probiotics. We find that the members of *B. clausii* species harbor high heterogeneity at the species as well as genus level. The genes conferring resistance to chloramphenicol, streptomycin, rifampicin, and tetracycline in the *B. clausii* ENTPro strain could be identified. The genes coding for the bacteriocin gallidermin, which prevents biofilm formation in the pathogens *Staphylococcus aureus* and *S. epidermidis*, were also identified. KEGG Pathway analysis suggested that the folate biosynthesis pathway, which depicts one of the important roles of probiotics in the host, is conserved completely in *B. subtilis* and minimally in *B. clausii* and other probiotics.

**Conclusions:**

We identified various antibiotic resistance, bacteriocins, stress-related, and adhesion-related domains, and industrially-relevant pathways, in the genomes of these probiotic bacteria that are likely to help them survive in the harsh gastrointestinal tract, facilitating adhesion to host epithelial cells, persistence during antibiotic treatment and combating bacterial infections.

## Background

Probiotics are live microbes which when consumed in sufficient amount helps to resume the original gut microflora, distressed by diarrhea or antibiotic intake [1]. Most bacterial probiotics such as *Lactobacillus* and *Bifidobacteria,* which are inhabitants of the gut, are available as lyophilized preparations of vegetative cells while some probiotic bacterial preparations that belong to the genus *Bacillus* are available in the form of spores [2]. Bacterial spores are dormant and resistant to heat, desiccation, dehydration and are extremely stable, which is a desirable property for probiotics [3, 4]. The spores of *Bacillus* germinate in the gut and the vegetative cells are vital for the human gut health [5]. *Bacillus subtilis* belongs to one of the most studied and explored family *Bacillaceae and* many of its strains are being used as probiotics since the 1990s. The role of *Bacillus* species ranges from probiotic nature of *B. subtilis*, *B. clausii*, *B. coagulans*, *B. pumilus* and other strains to biological control agents (*B. thuringiensis* and *B. sphaericus*), and pathogenicity (*B. anthracis* and *B. cereus*). Several strains are economically important (*B. subtilis*) whereas others have medical importance (*B. licheniformis*) [6]. Some *Bacillus* spp. are industrially-important and produce proteins such as alkaline proteases, xylanases, amylases, and cellulases [4, 7].

In 2001, some *B. subtilis* strains, which were used in the probiotics and soap industry, were reclassified as *B. clausii* [8]. *B. clausii* spores are marketed as the probiotic Enterogermina**®** which consists of four *Bacillus* strains O/C, N/R, SIN, and T that are resistant to Chloramphenicol, Novobiocin/Rifampicin, Neomycin/Streptomycin, and Tetracycline, respectively [9, 10]. Although these four strains are known to have been derived from a single penicillin-resistant strain, *B. subtilis* ATCC 9799 [9, 11], secretome analysis have revealed variation in the expression level of some of the secreted proteins [12]. Also, the O/C strain of *B. clausii* inhibits the cytotoxic effect induced by the *Clostridium difficile* and *B. cereus* toxins [13]. The intrinsic antibiotic resistance in probiotics is considered advantageous in cases of antibiotics-probiotics combination prescriptions to restore healthy gut [14, 15]. The mode of action of *B. clausii* as a probiotic is not clear, but the strains have been reported to secrete some proteins that are involved in the immunomodulatory mechanism, adaptation and their colonization in the human gastrointestinal tract (GIT) [12, 13, 16–18]. Uniquely, *B. clausii* harbors *erm(34)* gene that imparts the resistance to erythromycin. The *erm(34)* gene is not a homolog of *erm(A)*, *erm(B)*, *erm(C)* and *erm(TR)* genes for MLSB resistance in Gram-positive human pathogens and *erm(D)*, *erm(K)*, and *erm(J)* characterized in *B. licheniformis*, *B. halodurans*, and *B. anthracis,* respectively [19].

Various clinical trials and molecular studies [8, 13, 16, 20–23] have been performed to identify the major features that demarcate *B. clausii* probiotic strains from other *Bacillus* spp., but still, the genomic reasons of its probiotic activity have not been reported before. Therefore, we sequenced the composite genome sequence of *B. clausii* (composite of all four strains of *B. clausii* used in the probiotic formulation) from Enterogermina**®**, an oral probiotic, marketed by Sanofi in India. The composite genome obtained from the sequencing of this probiotics was named as *B. clausii* ENTPro. We performed an extensive analysis to identify the genomic features known to impart probiotic properties in *B. clausii* viz.*,* adhesion to gut, withstanding harsh conditions in the gut, antibiotic resistance, and biosynthesis pathways. In addition, to gain insight into the genomic features of different probiotics, we have compared pathways, types of bacteriocins and antibiotic resistance genes in different *Bacillus* probiotics.

## Results

### Genome features of *B. clausii* ENTPro

**Fig. 1:**
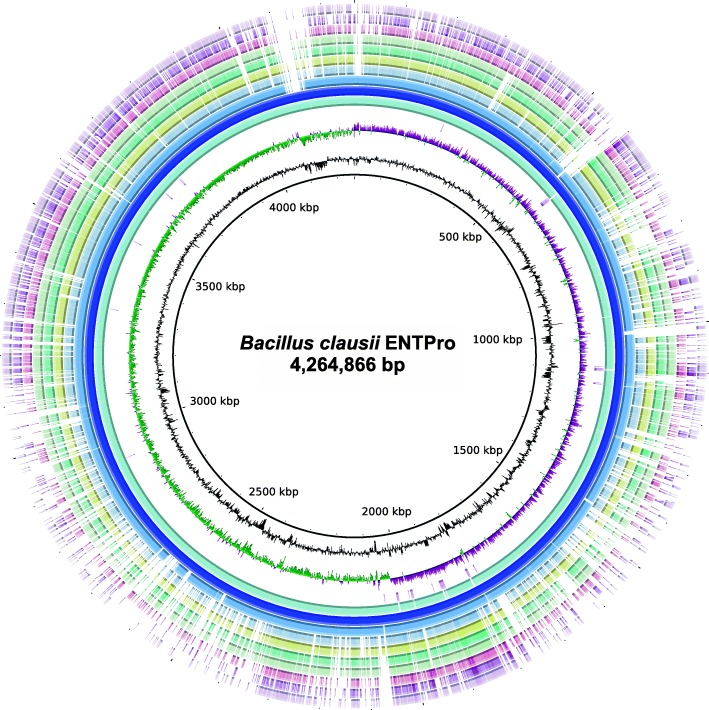
Circular representation of the *B. clausii* ENTPro composite genome. Here, *B. clausii* ENTPro was taken as reference genome and circles from inside to outside represents as: Circle 1 and 2 represent GC content and GC skew of *B. clausii* ENTPro, circle 3 represent encoded RNAs in *B. clausii* ENTPro; circle 4 represent genes encoded by *B. clausii* ENTPro; circle 5 depicts *B. clausii* ENTPro composite chromosome; Circle 6 depicts the mapping of *B. clausii* ENTPro genome against genome of *B. clausii* KSM-K16; further the circles 7 to 13 represents the genomes of *B. sp.* JCM 19045, *B. sp.* JCM 19046, *B. sp.* JCM 19047, *B. lehensis* G1, *B. halodurans* C-125, *B. cellulosilyticus* DSM 2522, *B. pseudofirmus* OF4 mapped on *B. clausii* ENTPro respectively. BRIG 0.95 was used to build the circular representation. Mapping studies were done using BLASTn with an E-value cut-off 1e^− 5^

De novo assembly of PacBio sequencing reads of *B. clausii* ENTPro gDNA resulted in two contigs: one long circular contig of 4,264,866 base pairs (bp) and one short circular 31,475 bp contig. The long contig represents the composite circular chromosome of *B. clausii* ENTPro with an average GC content of 44.75% **(**Fig. 1**)** and the smaller one (GC Content: 39.9%) is likely a plasmid. In addition, Illumina sequencing-based assembly resulted in 4.3 Mbp genome from 36 contigs and N50 of 344,696 bp, which overlaps completely with the genome assembled using PacBio reads. The composite genome obtained from PacBio sequencing reads was submitted to GenBank [NC_006582.1] and further used for all the comparisons in this study. *B. clausii* ENTPro genome is 99.8% similar to another probiotic strain *B. clausii* B106 [NFZO01] (Additional file 1: Figure S1A), followed by 94.3% similarity to *B. clausii KSM-K16* [NC_006582.1] (Additional file 1: Figure S1B), whereas other members of the same species are 50–94% similar. This suggests that the members of this species are quite diverse as characterized by their genome-genome distance calculator (GGDC) values (Additional file 1: Table S1). Our analysis suggests that probiotic strains within *B. clausii* such as ENTPro, B106, and UBBC-07 are highly similar to each other as compared to other strains.

The plasmid sequence is novel and does not have any close similarity with other plasmids in the NCBI nucleotide database (NT). Most of the proteins encoded by the plasmid sequence are hypothetical and are not functionally characterized. We mapped Illumina reads against the plasmid database downloaded from NCBI to identify if we could obtain hits to any previously known plasmids. Very few reads mapped on to known plasmids and no full plasmid could be retrieved using the Illumina reads. Therefore, we concluded that the identified plasmid sequence harbored by *B. clausii* ENTPro is novel.

Annotation of the *B. clausii* ENTPro genome revealed the presence of 4384 protein-coding sequences, which constitute 86.73% of the genome with an average length of 843 bp (ranging from 113 to 9509 bp) **(**Table 1**)**. A total of 1215 Coding DNA Sequences (CDS) were annotated as hypothetical proteins, accounting for 27.72% of the total proteins. The ENTPro genome has all the three proteins R (restriction), M (modification), and S (specificity) that belongs to the Type I RM system. m6A methylation was observed in > 96% of the motifs G**A**GNNNNNNRTGC and GC**A**YNNNNNNCTC in the genome at 2nd and 3rd positions, respectively. There are 75 tRNA genes and seven complete rRNA operons (> 99% identity) in the *B. clausii* ENTPro genome. 16S rRNAs obtained from the de novo assembly of *B. clausii* ENTPro genome shows 99.8% similarity with *B. clausii* Enterogermina strains O/C, T, N/R, and SIN. This is in line to previously known variations in 16S rRNA genes in bacterial genomes [24]. Most of the varying sites were present in the V1 region of the 16S rRNA sequences even in *B. clausii* KSM-K16 and *B. clausii* DSM 8716 (Additional file 1: Figure S2).

**Table 1:** Genome assembly and annotation statistics for *B. clausii* ENTPro composite genome

Chromosome genome assembly and annotation statistics of *B. clausii* ENTPro		
	Chromosome	Plasmid
Sequencing data	P6 polymerase and C4 [P6C4] Chemistry based PacBio sequencing	
Bio Project Number	PRJNA242453	
NCBI Accession number	CP012475	CP012476
Genome size (in bp)	4,264,866	31,475
GC content (%)	44.75	39.9
Chromosome/Contig	1	1
CDS	4384	40
% Coding sequences	86.73	84.45
CDS from (+) strand	2254	35
CDS from (−) strand	2130	5
Max. CDS length	9509	2711
Mean CDS length	843	664
Hypothetical proteins	1215	33
Hypothetical proteins (%)	27.72	82.5
tRNA	76	NA
rRNA	7 operons (21 rRNAs)	NA

**Fig. 2:**
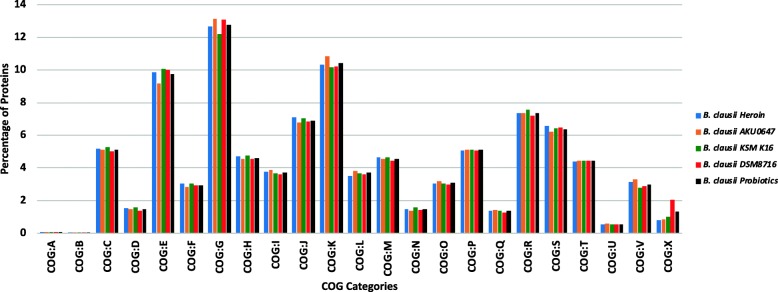
Cluster of Orthologous groups (COG) categories in *B. clausii* genomes. The X-axis represents the COG groups and the Y-axis represents the average number of proteins in respective COG groups. The genomes of *B. clausii* are clustered as per the properties. *B. clausii* Heroin represents all the organisms isolated from the Heroin samples [PRJNA395369]. *B. clausii* Probiotics represents all the probiotic strains as a single category. The COG categories are identified by capital letters as follows: A, RNA processing and modification; B, Chromatin structure and dynamics; C, energy production and conversion; D, cell cycle control, cell division and chromosome partitioning; E, amino acid transport and metabolism; F, nucleotide transport and metabolism; G, carbohydrate transport and metabolism; H, coenzyme transport and metabolism; I, lipid transport and metabolism; J, translation; K, transcription; L, replication; M, cell wall/membrane/envelope biogenesis; N, cell motility; O, post-translational modification, protein turnover, chaperones; P, inorganic ion transport and metabolism; Q, secondary metabolites biosynthesis, transport and catabolism; R, general function prediction only; S, function unknown; T, signal transduction mechanisms; U, intracellular trafficking and secretion; V, defense mechanisms; and X, Mobilome: prophages, transposons

Amongst the total proteome, ~ 75% (3311) proteins could be categorized into Clusters of Orthologous Groups (COGs) functional groups. Among these mapped proteins, ~ 35% belonged to the metabolism category, ~ 14% to cellular processes and signaling and ~ 16% proteins to information storage and processing. According to COG mapping data, 152 proteins are involved in signal transduction mechanisms (COG: T) and 44 proteins were reported to function in secondary metabolites biosynthesis, transport, and catabolism (COG: Q). COG assignments to proteomes of *B. clausii* members revealed that all the organisms have similar number of proteins assigned to various COG categories (Fig. 2).

### Phylogenetic position of *B. clausii* as inferred from housekeeping proteins-based phylogeny

**Fig. 3:**
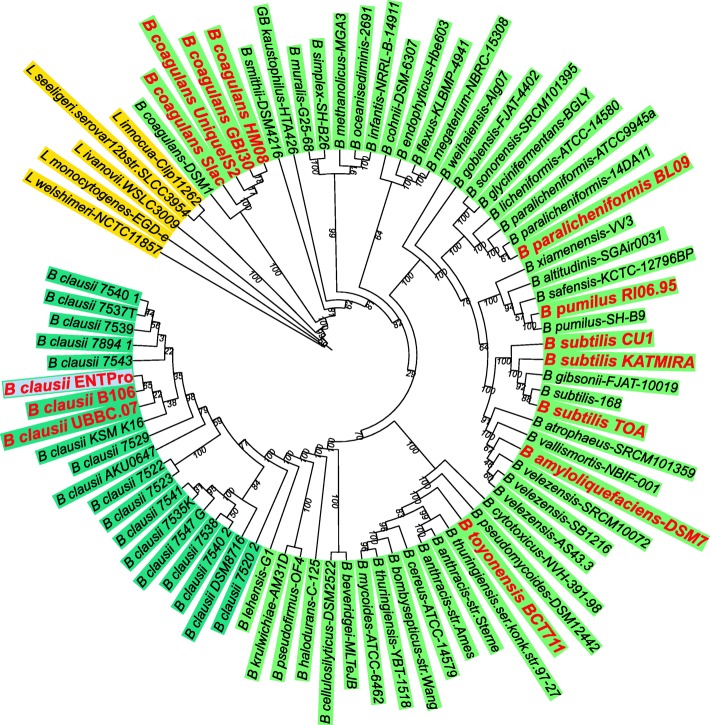
Housekeeping proteins based Maximum Likelihood phylogenetic tree The outgroups are colored with a yellow background, *Bacillus* species are colored with light green background and *B. clausii* members are colored with dark green background. The *Bacillus* probiotics are written with red labels with double the size of the rest of the organisms. Values on branches represent the bootstrap values.

Phylogenetically, *B. clausii* clustered in a separate clade with further grouping within this clade (Fig. 3). The phylogenetic tree reveals that ENTPro strain is closest to the B106 strain of *B. clausii*. Both these probiotic strains are further similar to another probiotic strain UBBC-07 of *B. clausii*. All these probiotic strains share a common ancestor with industrial *B. clausii* KSM-K16 strain. This phylogenetic placement of *B. clausii* probiotic strains is concordant with the whole genome similarity matrix as obtained by GGDC [25]. Other *B. clausii* “Heroin” strains form several different groups within the *B. clausii* clade. Interestingly, the *B. clausii* proteome matches the proteome of other *Bacillus* species at < 70% identity. This clearly suggests the genomic heterogeneity of *B. clausii* genome in comparison to other *Bacillus* species. We also included all *Bacillus* probiotics genomes in phylogenetic analysis to investigate their position phylogenetically [26]. *Bacillus* probiotics shared clades with their species members. Interestingly, probiotic strains cluster together e.g. *B. clausii*, *B. coagulans* and *B. subtilis*.

### *B. clausii* ENTPro as a derived strain from four different strains

*B. clausii* Enterogermina**®** is a mixture of four different strains, each of which is supposed to confer resistance against specific antibiotics, namely novobiocin and rifampicin (strain N/R), chloramphenicol (strain O/C), streptomycin and neomycin (strain SIN) and tetracycline (strain T) [12]. The specific genes conferring resistance could not be traced in the literature so different in silico strategies were employed to identify possible genes that could help impart resistance to these antibiotics in *B. clausii* ENTPro (Table S2 and S3).

*Rifampicin:* Rifampicin resistance is acquired by specific mutations at positions 516, 526 and 531 in the *rpoB* gene in *Escherichia coli* [27]. These mutations are mapped in the center of the *rpoB* gene in 3 regions: one cluster covering 507–533 amino-acid (AA); cluster II covering AA 563–572 and cluster III with AA change at position 687, which altogether are referred to as RIF resistance determining region (RRDR) [27]. In order to find the presence of RRDR region in RpoB protein in ENTPro, the RpoB protein sequences from all *Bacillus* spp. were retrieved and aligned with *E. coli* RpoB protein sequence [Accession Number: NP_418414.1]. P_524_- > L (corresponding to 567 AA position in *E. coli* RpoB protein sequence) AA change was observed in *B. clausii* ENTPro strain that was not observed in other *Bacillus* spp. (Additional file 1: Figure S3).

*Chloramphenicol:* Chloramphenicol acetyltransferase, involved in conferring resistance against chloramphenicol [28], was identified from the proteome of *B. clausii* ENTPro [Accession Number: WP_035203840.1].

*Streptomycin:* Pfam domains, known to impart resistance against streptomycin, were identified in *B. clausii* ENTPro. Nine proteins in *B. clausii* had the Pfam domain PF02522, PF01636, PF01909, PF04439, PF04655, PF07091, PF07827, and PF10706 that has core domain aminoglycoside. Two proteins had streptomycin adenylyltransferase domain (PF04439), six proteins have aminoglycoside phosphotransferase [PF01909] domain and one protein has Kanamycin nucleotidyltransferase [PF07827] domain in their sequence (Additional file 1: Table S2). This suggests the presence of domains that are involved in imparting resistance to streptomycin. In addition, the Kyoto Encyclopedia of Genes and Genomes (KEGG) pathways analysis of the organism reveal the presence of complete KEGG pathway for the streptomycin biosynthesis in the *B. clausii* ENTPro (Additional file 1: Figure S4).

**Fig. 4:**
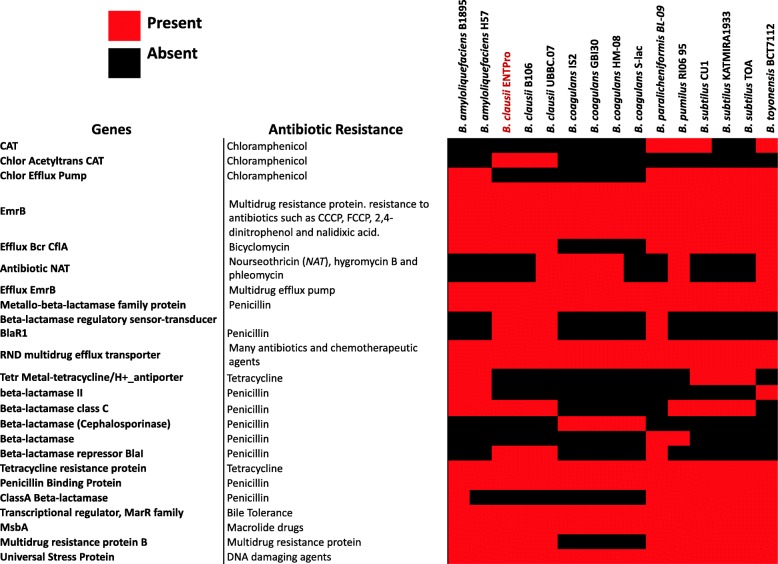
The binary matrix of Antibiotic resistance in *Bacillus* probiotics. Red color fill marks the presence whereas black color represents the absence of antobiotic resistance genes. The rows represent the name of the Antibiotic resistance categories and the columns are the *Bacillus* probiotics

*Tetracycline:* The domains conferring tetracycline resistance [RF0133, RF0134, RF0135, and RF0127] were present in *B. clausii* ENTPro (Fig. 4). The presence of these genes in the composite genome of *B. clausii* ENTPro was further confirmed by mapping the Illumina reads to these genes.

### Probiotic properties of *B. clausii* ENTPro

Probiotics are beneficial components of microbiota that modulates immunological, respiratory and gastrointestinal functions [29]. For imparting these functions probiotics adhere to the mucosal membrane to interact with the host and have acidic, alkaline and oxidative stress resistance and stress adaptation proteins [6]. Probiotics are believed to have good adherence capacity, which promotes gut residence time, pathogen elimination and adhesion to the epithelial layer of host cells and exerting immune modulation.

Pfam analysis reveals the presence of three proteins involved in adhesion namely a mucus-binding protein with ‘Gram_pos_anchor’ Pfam domain [PF00746] at the C-terminus, a collagen-binding protein with LPXTG motif at the C-terminus and a fibronectin-binding protein [30] (Additional file 1: Table S2). These adhesion proteins may help facilitate the probiotic bacterium to bind and help in the direct interactions with the intestinal mucosa layer.

Probiotic *B. clausii* has to encounter various harsh environmental conditions during transit in the GIT such as the acidic environment in the stomach, bile juice environment in the small intestine, oxidative stress, and osmotic stress [1]. When a bacterium faces an acidic environment, H^+^ homeostasis is maintained by F0F1 ATP synthase pump, which work by hydrolyzing ATP to pump protons (H^+^) from the cytoplasm [1, 31]. We found that this synthase complex is present in ENTPro genome as a full operon [DB29_02342--DB29_02349].

The bacteria have to face the toxicity of bile salts that induce intracellular acidification and act as detergents that disrupt biological membranes [32]. Five proteins were identified that were involved in bile tolerance mechanism; two belong to ornithine decarboxylase [33] and three to sodium bile acid symporter family [34, 35] (Additional file 1: Table S2).

*B. clausii* ENTPro also harbors general stress adaptation proteins. The universal stress protein UspA [PF00582] is important for survival during cellular growth arrest and reprograms the cell towards defense and escape during cellular stress [36, 37]. Molecular chaperones that may impart resistance against environmental stress were obtained through annotation and Pfam domain search such as the chaperonin GroES [PF00166] and GroEL [38, 39] and one heat shock protein 33 [PF01430], two copies of cold shock proteins CspA [PF00313], three Clp protease [PF00574] and HtpX and HrcA-like heat shock proteins. These proteins play an important role in basic cellular functions that includes growth, the stability of DNA and RNA and they also prevent the formation of inclusion bodies [40–42].

For hyperosmotic stress and heat resistance, *B. clausii* ENTPro harbors one copy each of the chaperone protein DnaJ [PF00226] and nucleotide exchange factor GrpE [PF01025]. Also, two methionine sulfoxide reductase A [43] [PF01625] were present in *B. clausii* ENTPro that provides resistance in oxidative stress (Additional file 1: Table S2). This suggests that *B. clausii* ENTPro has proteins to improve adhesion and handling stress and harsh conditions in the human gut.

### Antibiotic resistance in *Bacillus* probiotics

Antibiotic resistance is a common phenomenon in Gram-positive bacteria [44–46]. It is accomplished by genes acquired either horizontally through plasmids, or foreign DNA recombination, or mutations at different chromosomal loci in the bacterial genome [47]. It is preferred that probiotic strains carry few antibiotic resistance genes as possible so that they are not a putative source for transferring these genes to other gut bacteria including pathogens [46]. However, on the other hand since some of these probiotics are administrated alongside antibiotics, some resistance to commonly administrated antibiotics are desirable.

Presence of a novel plasmid sequence in *B. clausii* ENTPro could be a possible source of antibiotic-resistance gene transfer but we could not identify any potential antibiotic-resistance domain(s) in the plasmid. We also searched for the presence of antibiotic resistance genes and efflux pumps in the genomes with multiple methods to avoid false positives.

The Chloramphenicol acetyltransferase, that confers resistance against chloramphenicol, is absent in *B. amyloliquefaciens* and *B. coagulans* whereas chloramphenicol efflux pump was present in *B. amyloliquefaciens* (Fig. 4). This would imply the presence of chloramphenicol resistance in all the *Bacillus* probiotics except *B. coagulans*. Different classes of beta-lactamase were present in one or the other *Bacillus* probiotics that clearly suggest the presence of resistance against Penicillin in all the *Bacillus* probiotics. Multidrug resistance protein, a universal stress protein, EmrB, and its efflux pump, tetracycline resistance protein, and penicillin-binding protein are present in all the *Bacillus* probiotics. This suggests that most of the *Bacillus* probiotics are resistant to common antibiotics.

Erythromycin resistance was identified by subjecting the *erm(34)* gene sequence (GenBank Identifier: AY234334) of *B. clausii* DSM8716 to BLASTn against all *Bacillus* genomes. This gene was identified in *B. clausii* ENTPro named as “SSU rRNA (adenine (1518)-N (6)/adenine (1519)-N (6))-dimethyltransferase” (GenBank Identifier: ALA53582). The gene was also identified in all the *B. clausii* genomes. The gene sequence shared 61% identity to rRNA adenine methyltransferase of *B. halodurans* and 57% identity to rRNA adenine methyltransferase of *B. licheniformis*, *B. anthracis*, *B. sonorensis* and *B. fordii*. The rRNA adenine methyltransferase gene from other *Bacillus* spp. shared 20–50% identity with *erm(34)* gene. The result reveals that the *erm(34)* gene is unique to *B. clausii* and is not present in other members of the *Bacillus* genus.

Vancomycin resistance, as observed from KEGG pathway analysis, (Additional file 1: Figure S5) was identified only in *B. toyonensis* while absent in other *Bacillus* probiotics. The accessory proteins of vancomycin resistance operon were present in some of the *Bacillus* probiotics, but resistance-conferring genes were completely absent.

We would like to add an advisory note that previous studies have shown that an organism may exhibit intrinsic resistance to a few antibiotics that could not be related to its genotype [46]. Though we have endeavored to relate the genome-level occurrence of antibiotic resistance proteins or domains to their probable phenotypes, we have not performed any phenotypic studies to substantiate these analyses and/or confirm for intrinsic resistance. Further, the current situation may constitute a safety concern because of the possibility of transfer of antibiotic gene transfer to gut flora [46].

### Bacteriocins in *Bacillus* probiotics

**Fig. 5:**
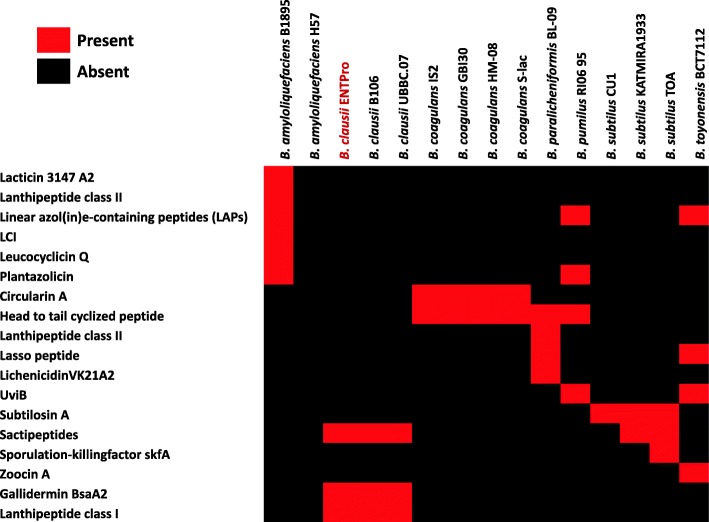
The binary matrix of Bacteriocins in *Bacillus* probiotics. Red color marks the presence of the bacteriocin whereas black color represents absence of bacteriocins. The rows represent the name of the bacteriocins, and the columns are the *Bacillus* probiotics

Bacteriocins are proteinaceous toxins produced by bacteria that act as narrow-spectrum antibiotics to inhibit the growth of similar or closely related bacterial strains [48, 49]. They can help probiotics to survive the toxins produced by invading bacteria by inhibiting their growth and hence can result in beneficial effects on the hosts. The identified bacteriocins in all the probiotics are represented in a presence-absence binary matrix in Fig. 5. Several of these bacteriocins are already well utilized in therapeutics [50] and their spectrum against pathogens is well established [9, 51–53].

Gallidermin identified via in silico analysis in *B. clausii* genomes is known to efficiently prevent biofilm formation in the pathogens *S. aureus* and *S. epidermidis* species [26]. This bacteriocin has also been reported to be effective in skin disorders including acne, eczema, folliculitis, and impetigo where the targets organisms are Propionibacteria, Staphylococci, and Streptococci [50].

Lacticin 3147 A2 and Leucocyclin Q as identified in *B. amyloliquefaciens* are broad-spectrum bacteriocins. Lacticin has been used effectively in the treatment of bacterial mastitis, Staphylococcal and Enterococcal infections including vancomycin-resistant *Enterococci* [50] and is effective against *Listeria* infections [51]. Similarly, leucocyclicin Q exhibit bactericidal or bacteriostatic effects on Gram-positive bacteria, including food-borne pathogens, such as *Lactococcus*, *Weissella paramesenteroides*, *Pediococcus dextrinicus*, *Enterococcus*, *Streptococcus*, and *Leuconostoc* [52]. Plantazolicin identified in *B. amyloliquefaciens* and *B. pumilus* has nematicidal activity [54]. Cirucularin A produced by *B. coagulans* has been reported to be the most effective bacteriocin against *C. tyrobutyricum* NIZOB570, a known cheese-spoilage bacterium [55] and also Lactococci*,* Enterococci, and some *Lactobacillus* strains [56]. LichenicidinVK21A2 identified in *B. paralicheniformis* is considered as self-immunity bacteriocin that exhibits antimicrobial activity against several strains of *Listeria monocytogenes*, methicillin-resistant *S. aureus*, and vancomycin-resistant Enterococcus [57]. Zoocin A in *B. toyonensis* shows antimicrobial activity against several other Streptococci by cleaving the peptidoglycan cross-links of the target cell wall [58].

Subtilosin A produced by *B. subtilis* is also a broad range bacteriocin that is effective against *Listeria monocytogenes*, and strains of *E. faecalis*, *P. gingivalis*, *K. rhizophila*, *Enterobacter aerogenes*, *Streptococcus pyogenes,* and *Shigella sonnei* [53]. Sporulation-killing factor skfA produced by *B. subtilis* induces the lysis of other *B. subtilis* cells that have not entered the sporulation pathway. This cannibalistic behavior provides a source of nutrients to support those cells that have entered sporulation [59, 60]. At high concentrations, it can also inhibit the growth of other bacteria [61]. The presence of well-characterized bacteriocins in the *Bacillus* probiotics suggests their important role in fighting against the pathogen in the gut.

### Folate biosynthesis pathways in *Bacillus* probiotics

**Fig. 6:**
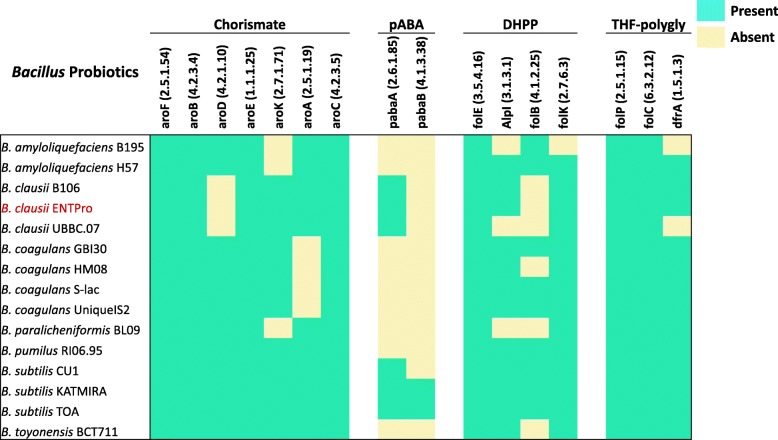
The binary matrix of components of folate biosynthesis pathways in *Bacillus* probiotics. Sea green marks the presence whereas yellow is absent. The rows represent the name of the *Bacillus* probiotics whereas column represents the pathway components

The gut microbiota aids the host, playing a crucial role in nutrient digestion and energy recovery. Due to potentially relevant applications, the capacity to yield folate has been investigated in various probiotic strains. Previously, the presence of these pathways was reported in *Lactobacillus* and *Bifidobacterium* probiotics but was not explored in *Bacillus* probiotics [62] except *B. subtilis* [63]. We performed the identification of key components of folate production pathways in *Bacillus* probiotics using KEGG Pathway database [64]. The analysis of genome sequences of *Bacillus* probiotics revealed the presence of complete operon to synthesize para-aminobenzoic acid (PABA) de novo only in *B. subtilis* probiotics (Fig. 6). On the other hand, the enzymes, necessary for chorismate conversion into PABA are present in almost all the *Bacillus* probiotics. Moreover, the shikimate pathway for chorismate production is complete only in *B. subtilis*, *B. pumilus* and *B. toyonensis*, while the pathway’s component are partially present in all the other *Bacillus* probiotics. On the other hand, *Bacillus* probiotic strains contain the genes of DHPPP de novo biosynthetic pathway, the gene encoding dihydropteroate synthase (EC 2.5.1.15) and gene encoding dihydropteroate synthase (EC 2.5.1.15). Therefore, it is expected that these strains are not auxotrophic for folates or DHP but can produce folate in the presence of PABA supplementation. The presence/absence of the components of the folate biosynthesis pathway is reported based on KEGG pathway analysis. Previous studies have revealed that *B. subtilis* genome harbor all the pathways components and have been engineered for folate production [63, 65, 66].

## Discussion

In this study, we report the complete composite genome of *B. clausii* ENTPro, sequenced from a commercially available probiotic, Enterogermina**®** that is a mixture of four closely related strains. Oral probiotics are popularly recommended by physicians as an adjunct to antibiotic therapy to avoid antibiotic-induced diarrhea and/or other gastrointestinal distress. We determined the composite genome of *B. clausii*, to identify the features responsible for its probiotic properties and correlate it to the phenotypic properties mentioned previously in the literature [26, 50, 61, 67–71]. While literature studies also mention the presence of four closely related strains in the probiotic Enterogermina**®,** [2, 12, 72] our single chromosome assembly suggests that the different strains are inseparable. However, variations in number of bacteria from each strain in the formulation has also been reported previously [73].

Our investigation revealed the presence of antibiotic resistance genes that *B. clausii* ENTPro harbors. However, we could not find the genes conferring Novobiocin and Neomycin resistance, possibly because the genome reported here is a composite of the four strains. The possibility of the identified genome to be strain-specific could explain these missed antibiotic resistance genes. Previously reported secretome analysis [12] supports the presence of four related strains, and our genome data suggests that they are very closely related.

COG assignments of all the *B. clausii* strains did not show any differences in probiotic strains versus industrial strains of *B. clausii*. Apart from these common features within the members of *B. clausii* genus, the low genome-genome distance revealed that its members are quite diverse. We further used phylogenetic methods to understand the relationship of the members of *B. clausii* and other *Bacillus* using representatives of each species. The phylogenetic tree clearly has a separate group where members of *B. clausii* lies with *B. lehensis*, *B. halodurans* whereas *B. subtilis, B. coagulans* formed separate clades. The *B. clausii* proteome analysis also supported its distant placement in the phylogenetic tree from other *Bacillus* members. These results indicate that *B. clausii* is unique in comparison to other *Bacillus* species.

Using in silico mining approaches, we previously reported the sporulation proteins as well as various other proteins that might play a role in probiotic function such as molecular chaperones, stress proteins, flagellin, and mucin binding protein in two other *Bacillus* probiotics marketed in India namely *B. coagulans* S-lac and *B. subtilis* TOA JPC [67]. We also reported the presence of adhesins, which might aid in adhesion to the mucosal layer of host tissues. All these proteins are present in the currently compared probiotic genomes as well. In addition to these, in the current study, we looked for the antibiotic resistance genes, bacteriocins, and folate biosynthesis pathways. Multiple strategies were used in our analysis to comprehensively catalog these domains, nevertheless, these bacteria may harbor genes that confer intrinsic antibiotic resistance. For example, the presence of chloramphenicol, tetracycline and vancomycin resistance in *B. toyonensis* has been reported previously [68, 69]. Bile tolerance is reported for *B. clausii* Enterogermina in a recent study [74] that substantiates our findings. We have also explored the occurrence of bacteriocins in different *Bacillus* probiotics. The presence of different bacteriocins makes these organisms unique. For example, *B. clausii* would be effective in *S. aureus* infections [26] in skin disorders [50] while *B. amyloliquefaciens* in food industry especially yogurt beverage and probiotic [70], while *B. toyonensis* shows antimicrobial activity against several Streptococci [58, 75, 76].

One of the important roles of probiotics is nutrient digestion and energy recovery by producing folate. Folate occurs naturally in food [62] and it is required for the efficiency of DNA replication, repair and methylation process in humans [77, 78]. The folate-producing probiotic strains could possibly confer protection against cancer, inflammation, cardiovascular disease, stress, and depression [62, 63, 77, 78]. This role of probiotics has been extensively studied for their commercial utilization in folate production [62, 79]. We investigated the pathways involved in the production of folic acid in these probiotics. Not surprisingly, we found all the pathway components intact in *B. subtilis* which is being engineered for the enhanced synthesis of folate [63]. Some core components of the folate synthesis pathways were present in other *Bacillus* probiotics, suggesting that they may also be potential sources for the de novo synthesis of folate.

The composite genome of *B. clausii* ENTPro and the comparative analysis presented here resulted in identification of several genes and pathways of interest required for probiotic action. This study can serve as a starting point for the experimental characterization of these gene products and bacteriocins in order to obtain a deeper understanding into the mechanism of probiotic action of these important microbes.

## Conclusions

The composite circular genome of *B. clausii* strain ENTPro, isolated from Enterogermina**®**, an oral probiotic, marketed by Sanofi in India, is reported. The genomes of different strains are inseparable as suggested by complete circular assembly using long PacBio reads. *B. clausii* ENTPro shares high similarity with probiotic strains of genus *B. clausii* whereas it is quite diverse as compared to other *Bacillus* probiotics. The ML tree based on 25 housekeeping protein sequences, clearly places *B. clausii* in a separate clade as an outgroup of the *Bacillus* species.

In this study, we report the genes that are responsible for conferring antibiotic resistance in *B. clausii*. We could identify all the antibiotic resistance genes that are indicative of the presence of all four strains in the assembled composite genome. Also, we compared the presence of antibiotic resistance-conferring genes and related pathways in all the probiotic *Bacillus* genomes. The most important finding of our study is the identification of bacteriocins in *Bacillus* probiotic genomes, which could be directly related to their usage in food and beverage industry. For e.g. gallidermin bacteriocin identified in *B. clausii*, functions against *S. aureus* biofilm formation and infections [26, 80]. The bacteriocins in *B. amyloliquefaciens* fight against foodborne pathogens, [81] which clearly supports its usage in the yoghurt beverage industry. *B. paralicheniformis* secretes bacteriocins to prevent *Listeria*, *S. aureus,* and *Enterococcus* borne infections [57, 82].

The other important aspect we studied was the presence of genes necessary for the production of folate. We found that *B. subtilis* can produce folate de novo whereas other *Bacillus* probiotics depend on supplements viz. pABA to produce the same. Several important components and alternative pathways for folate production were present in other *Bacillus* probiotics but not complete like in *B. subtilis*. While identifying several genes and pathways of interest is insufficient to explain the concerted probiotic action, we believe our study shed light on several genomic aspects of different *Bacillus* probiotics. We trust that the comparative genomics analysis presented here will pave the way for experimental characterization of our findings, and to possibly engineer these organisms for enhanced probiotic actions.

## Methods

### Isolation and purification of *B. clausii* genomic DNA

*B. clausii* spore suspension drug “Enterogermina**®**, Sanofi-Aventis” (Batch No. 120965; Mfd. 12/2011 and Exp. 11/2013) was procured from a drugstore in Chandigarh, India and was cultured in March 2013. Bacterial cells were suspended in Milli-Q water, serially diluted, and plated on ATCC medium: 688 nutrient agar plates. The plates were incubated at 25 °C for 48 h. DNA isolation was performed using the ZR Fungal/Bacterial DNA miniprep kit (Zymogen) as per instructions in its user manual. After isolation, the genomic DNA was treated with RNase A (1 μl of a 10 μg/mL stock solution for 100 μl of a solution containing DNA) and incubated at 37 °C for 30 min. Then, 1/10 volume of 3 M sodium acetate (pH 5.2) and 2.5 volumes of absolute ethanol was added followed by incubation at − 20 °C overnight and centrifugation at 14,000 rpm for 30 min at 4 °C. The supernatant was carefully discarded; the pellet was rinsed with 70% ethanol and centrifuged again at 14,000 rpm for 15 min at 4 °C. The ratio of OD at 260/280 nm was > 1.8 as observed by NanoDropND-1000 spectrophotometer.

### Genome sequencing

#### PacBio sequencing

The probiotic *B. clausii* ENTPro was sequenced using PacBio P6C4 chemistry at Genome Quebec Centre, McGill University. DNA samples were sheared and concentrated using AMPure magnetic beads and treated by ExoVII to remove single-stranded ends. SMRTbell libraries were created using the ‘Procedure and Checklist–20 kb Template Preparation Using BluePippin™ Size Selection System protocol. Size Selection was performed to retain longer reads (> 10 k reads) for sequencing. Blunt ligation reactions were prepared and SMRTbell templates were purified using AMPure magnetic beads. BluePippin™ The Size-selected SMRTbell templates were annealed and polymerase was added for Sequencing. Single SMRT cell was run on the PacBio RS II system using P6C4 chemistry and a 180-min data collection mode.

#### Illumina sequencing

*B. clausii* ENTPro was also sequenced using the Illumina HiSeq PE platform. The library preparation was carried out according to the TruSeq DNA sample preparation protocol (Illumina, Inc., San Diego, CA) at C-CAMP, Bangalore, India. One μg of bacterial DNA was sheared to an average length of 300 to 400 bp. End repair, A-tailing, and adapter ligation (~ 120 base adapter) procedure was performed according to paired-end DNA sample preparation kit (Illumina, Size selection of adapter-ligated DNA was done in a range of 400 to 550 bases for DNA library). The insert size was taken in a range of 280 to 430 bases for DNA library. PCR enrichment was performed for eight cycles, and the samples were validated on a Bioanalyzer. Libraries were sequenced in a paired-end 100 base run, using TruSeq PE Cluster Kit v3-cBot-HS for cluster generation on C-bot and TruSeq SBS Kit v3-HS (Catalog No.: PE-401-3001) for sequencing on the Illumina HiSeq 1000 platform according to recommended protocols.

### Genome assembly and annotation

The PacBio reads were assembled de novo using Hierarchical Genome Assembly Process (HGAP) v2.0 [71] in SMRT portal using default parameters. Functional annotation was carried out by RAST (Rapid Annotation using Subsystem Technology) [83, 84], tRNA was predicted by tRNAscan-SE 1.23 [85] and rRNA genes by RNAmmer 1.2 [86]. The taxonomic characterization of the contigs was performed by subjecting the contigs to BLASTn [87] against NT database. The methylome was deduced by RS Modification and Motif analysis in SMRT portal (https://github.com/PacificBiosciences). The plasmid sequence was confirmed by plasmidSPAdes [88].

### Phylogenetic analysis

The *Bacillus* genus comprises more than 1000 genomes. To be efficiently able to plot the phylogenetic position of *B. clausii* in *Bacillaceae* family, we retrieved the *Bacillus* genomes that have been classified as representative genomes by NCBI (Accession Numbers in Additional file 1: Table S4). The representative genomes from genus *Listeria* and *Clostridium* were selected as outliers. Twenty five housekeeping proteins (ribosomal protein S11, ribosomal protein S13, ribosomal protein S19, ribosomal protein S2, ribosomal protein S3, ribosomal protein S5, ribosomal protein S9, ribosomal protein L11, ribosomal protein L13, ribosomal protein L19, ribosomal protein L2, ribosomal protein L20, ribosomal protein L27, ribosomal protein L3, ribosomal protein L4, ribosomal protein L5, ribosomal protein L6, ribosomal protein L7/L12, CTP synthase, DNA gyrase subunit B, DNA mismatch repair protein MutS, DNA primase, elongation factor Ts, elongation factor Tu, Transcription termination protein NusA) were retrieved from all the genomes and were concatenated. The sequences were aligned using Muscle [89] and the phylogenetic inference was drawn using Maximum likelihood [ML] approach based on PROTGAMMA model in RAxML [90] (Bootstrap: 100).

### Comparative genomics

For comparative analysis, all *B. clausii* genomes available on March 2018 were downloaded from NCBI. In addition, the genomes with reported probiotics properties were downloaded from the NCBI for comparison. All these genomes were annotated again using the RAST server [83] to remove the bias from different annotation strategies. COGs were identified by subjecting the proteomes of these organisms to BLASTp against COG database [91] at E-value 1e-5.

### Identification of genome features contributing to probiotic properties of *B. clausii* ENTPro

*B. clausii* ENTPro proteome was scanned using Hidden Markov Model (HMM) [92, 93] for the presence of specific domains involved in acid tolerance, adhesion, antibiotic resistance, antimicrobial production, heavy metal resistance, bile resistance, oxidative and universal stress resistance, and riboflavin synthesis.

#### Identification of antibiotic resistance genes

The Comprehensive Antibiotic Resistance Database (CARD) [94] and Pfam domains were downloaded and hmmscan [93] was run locally against the proteome of all the organisms to identify the domains that could impart antibiotic resistance. Rifampicin resistance was identified based on the mutation in *RpoB* genes. Chloramphenicol resistance was identified based on the presence of chloramphenicol acetyltransferase gene in the proteome of the respective organisms. The presence of *erm* (34) was found by subjecting its gene sequence to BLASTn against the NR database. Streptomycin biosynthesis and Vancomycin resistance were identified from the pathway analysis against the KEGG database [64].

#### Identification of Bacteriocins

Bacteriocins were reported as per the identification from the BAGEL3 server [95]. Any bacteriocin is considered present in a species if that bacteriocin is present at least in one more strain of that species.

#### KEGG pathway analysis

The proteomes of all the organisms were subject to bidirectional best hits to KEGG database [64, 96] to identify the components of folate biosynthesis pathways, streptomycin biosynthesis, and vancomycin resistance pathways.

## Supplementary information

**Additional file 1: Figure S1A.** Mauve alignment of *B. clausii* ENTPro with *B. clausii* B106 **Figure S1B.** Mauve alignment of *B. clausii* ENTPro with *B. clausii* KSM K-16. **Figure S2.** The 16S rRNA alignment of operons identified in *B. clausii* ENTPro and its parental strains. The V-regions are identified using V-Xtractor v. 2.1 67. The V regions are marked by red bars and mutated positions are marked in pink color. **Figure S3.** RpoB sequence alignment between *E. coli* and *B. clausii* genomes. **Figure S4.** The Streptomycin biosynthesis pathway components present in *B. clausii*. Green box marks the presence of enzymes present in *B. clausii*. **Figure S5.** The Vancomycin resistance pathway components present in *B. toyonensis* BCT-7112. Green box marks the presence of gene whereas the not filled box marks the absence. **Table S1.** Genome Similarity of *B. clausii* ENTPro with other *B. clausii* genomes. Formula I, II and III represents different methods used by GGDC to calculate the similarities. **Table S2.** Distribution of proteins involved in probiotic properties in *B. clausii* ENTPro genome. **Table S3.** Proteins and domains involved in conferring antibiotic resistance in *B. clausii* ENTPro. **Table S4.** Accession numbers of the genomes used to generate the phylogenies.

## Data Availability

This Whole Genome Shotgun project for chromosome and plasmid of *Bacillus clausii* ENTPro has been deposited at DDBJ/EMBL/GenBank under the accession CP012475 and CP012476.

## Abbreviations

AA: Amino-acid
bp: Base pairs
CDS: Coding DNA Sequences
COGs: Clusters of Orthologous Groups
GGDC: Genome-Genome Distance Calculator
GIT: Gastrointestinal Tract
KEGG: Kyoto Encyclopedia of Genes and Genomes
M: Modification
NT: Nucleotide database
PABA: Para-aminobenzoic acid
R: Restriction
RRDR: RIF resistance determining region
S: Specificity

## References

[1] Fuller R (1989). Probiotics in man and animals. J Appl Bacteriol.

[2] Cutting SM (2011). *Bacillus* probiotics. Food Microbiol.

[3] Nicholson WL, Munakata N, Horneck G, Melosh HJ, Setlow P (2000). Resistance of *Bacillus* endospores to extreme terrestrial and extraterrestrial environments. Microbiol Mol Biol Rev.

[4] Casula G, Cutting SM (2002). *Bacillus* probiotics: spore germination in the gastrointestinal tract. Appl Environ Microbiol.

[5] Kim SG, Becattini S, Moody TU, Shliaha PV, Littmann ER, Seok R (2019). Microbiota-derived lantibiotic restores resistance against vancomycin-resistant Enterococcus. Nature.

[6] Senok AC, Ismaeel AY, Botta GA (2005). Probiotics: facts and myths. Clin Microbiol Infect.

[7] Porwal S, Lal S, Cheema S, Kalia VC (2009). Phylogeny in aid of the present and novel microbial lineages: diversity in *Bacillus*. PLoS One.

[8] Senesi S, Celandroni F, Tavanti A, Ghelardi E (2001). Molecular characterization and identification of *Bacillus clausii* strains marketed for use in oral bacteriotherapy. Appl Environ Microbiol.

[9] Ciffo F (1984). Determination of the spectrum of antibiotic resistance of the “Bacillus subtilis” strains of Enterogermina. Chemioterapia.

[10] Mazza P, Zani F, Martelli P (1992). Studies on the antibiotic resistance of *Bacillus subtilis* strains used in oral bacteriotherapy. Boll Chim Farm.

[11] Green DH, Wakeley PR, Page A, Barnes A, Baccigalupi L, Ricca E (1999). Characterization of two *Bacillus* probiotics. Appl Environ Microbiol.

[12] Lippolis R, Siciliano RA, Mazzeo MF, Abbrescia A, Gnoni A, Sardanelli AM (2013). Comparative secretome analysis of four isogenic *Bacillus clausii* probiotic strains. Proteome Sci.

[13] Ripert G, Racedo SM, Elie A-M, Jacquot C, Bressollier P, Urdaci MC (2016). Secreted compounds of the probiotic *Bacillus clausii* strain O/C inhibit the cytotoxic effects induced by *Clostridium difficile* and *Bacillus cereus* toxins. Antimicrob Agents Chemother.

[14] Varankovich NV, Nickerson MT, Korber DR (2015). Probiotic-based strategies for therapeutic and prophylactic use against multiple gastrointestinal diseases. Front Microbiol.

[15] Hammad AM, Shimamoto T (2010). Towards a compatible probiotic-antibiotic combination therapy: assessment of antimicrobial resistance in the Japanese probiotics. J Appl Microbiol.

[16] Lopetuso LR, Scaldaferri F, Franceschi F, Gasbarrini A. *Bacillus clausii* and gut homeostasis: state of the art and future perspectives. Expert Rev Gastroenterol Hepatol. 2016:1–6. 10.1080/17474124.2016.1200465. 10.1080/17474124.2016.1200465 27291780

[17] Pradhan B, Guha D, Ray P, Das D, Aich P (2016). Comparative analysis of the effects of two probiotic bacterial strains on metabolism and innate immunity in the RAW 264.7 murine macrophage cell line. Probiotics Antimicrob Proteins.

[18] Patrone V, Molinari P, Morelli L (2016). Microbiological and molecular characterization of commercially available probiotics containing *Bacillus clausii* from India and Pakistan. Int J Food Microbiol.

[19] Bozdogan B, Galopin S, Leclercq R (2004). Characterization of a new erm-related macrolide resistance gene present in probiotic strains of *Bacillus clausii*. Appl Environ Microbiol.

[20] Marseglia GL, Tosca M, Cirillo I, Licari A, Leone M, Marseglia A (2007). Efficacy of *Bacillus clausii* spores in the prevention of recurrent respiratory infections in children: a pilot study. Ther Clin Risk Manag.

[21] Marseglia GL, Tosca M, Cirillo I, Licari A, Leone M, Marseglia A, Castellazzi AM, GC (2007). Efficacy of *Bacillus clausii* spores in the prevention of recurrent respiratory infections in children: a pilot study. Ther Clin Risk Manag.

[22] Lakshmi SG, Jayanthi N, Saravanan M, Ratna MS (2017). Safety assesment of *Bacillus clausii* UBBC07, a spore forming probiotic. Toxicol Rep.

[23] Canani RB, Cirillo P, Terrin G, Cesarano L, Spagnuolo MI, De Vincenzo A (2007). Probiotics for treatment of acute diarrhoea in children: randomised clinical trial of five different preparations. BMJ.

[24] Větrovský T, Baldrian P (2013). The variability of the 16S rRNA gene in bacterial genomes and its consequences for bacterial community analyses. PLoS One.

[25] Auch AF, von Jan M, Klenk H-P, Göker M (2010). Digital DNA-DNA hybridization for microbial species delineation by means of genome-to-genome sequence comparison. Stand Genomic Sci.

[26] Saising J, Dube L, Ziebandt A-K, Voravuthikunchai SP, Nega M, Götz F (2012). Activity of gallidermin on *Staphylococcus aureus* and *Staphylococcus epidermidis* biofilms. Antimicrob Agents Chemother.

[27] Goldstein BP (2014). Resistance to rifampicin: a review. J Antibiot.

[28] Schwarz Stefan, Kehrenberg Corinna, Doublet Benoît, Cloeckaert Axel (2004). Molecular basis of bacterial resistance to chloramphenicol and florfenicol. FEMS Microbiology Reviews.

[29] Floch MH, Walker WA (2011). Probiotics are considered nutritional supplements. J Clin Gastroenterol.

[30] Navarre WW, Schneewind O (1999). Surface proteins of gram-positive bacteria and mechanisms of their targeting to the cell wall envelope. Microbiol Mol Biol Rev.

[31] Cotter PD, Hill C (2003). Surviving the acid test: responses of gram-positive bacteria to low pH. Microbiol Mol Biol Rev.

[32] Begley M, Gahan CG, Hill C (2005). The interaction between bacteria and bile. FEMS Microbiol Rev.

[33] Azcarate-Peril MA, Altermann E, Hoover-Fitzula RL, Cano RJ, Klaenhammer TR (2004). Identification and inactivation of genetic loci involved with *Lactobacillus acidophilus* acid tolerance. Appl Environ Microbiol.

[34] Hagenbuch B, Dawson P (2004). The sodium bile salt cotransport family SLC10. Pflugers Arch - Eur J Physiol.

[35] Wong MH, Oelkers P, Craddock AL, Dawson PA (1994). Expression cloning and characterization of the hamster ileal sodium-dependent bile acid transporter. J Biol Chem.

[36] Nachin L, Nannmark U, Nystrom T (2005). Differential roles of the universal stress proteins of *Escherichia coli* in oxidative stress resistance, adhesion, and motility. J Bacteriol.

[37] Seifart Gomes C, Izar B, Pazan F, Mohamed W, Mraheil MA, Mukherjee K (2011). Universal stress proteins are important for oxidative and acid stress resistance and growth of *Listeria monocytogenes* EGD-e in vitro and in vivo. PLoS One.

[38] Ventura M, Canchaya C, Zink R, Fitzgerald GF, van Sinderen D (2004). Characterization of the groEL and groES loci in *Bifidobacterium breve* UCC 2003: genetic, transcriptional, and phylogenetic analyses. Appl Environ Microbiol.

[39] Susin MF, Baldini RL, Gueiros-Filho F, Gomes SL (2006). GroES/GroEL and DnaK/DnaJ have distinct roles in stress responses and during cell cycle progression in *Caulobacter crescentus*. J Bacteriol.

[40] Veinger L, Diamant S, Buchner J, Goloubinoff P (1998). The small heat-shock protein IbpB from *Escherichia coli* stabilizes stress-denatured proteins for subsequent refolding by a multichaperone network. J Biol Chem.

[41] Narberhaus F (2002). Alpha-crystallin-type heat shock proteins: socializing minichaperones in the context of a multichaperone network. Microbiol Mol Biol Rev.

[42] Jakob U, Gaestel M, Engel K, Buchner J (1993). Small heat shock proteins are molecular chaperones. J Biol Chem.

[43] Fu X, Adams Z, Liu R, Hepowit NL, Wu Y, Bowmann CF, et al. Methionine Sulfoxide Reductase a (MsrA) and its function in ubiquitin-like protein modification in *Archaea*. MBio. 2017;8. 10.1128/mBio.01169-17. 10.1128/mBio.01169-17 PMC5587910 28874471

[44] Marshall B, Petrowski D, Levy SB, Summers AO (1990). Inter- and intraspecies spread of *Escherichia coli* in a farm environment in the absence of antibiotic usage. Proc Natl Acad Sci U S A.

[45] Gibson MK, Forsberg KJ, Dantas G (2015). Improved annotation of antibiotic resistance determinants reveals microbial resistomes cluster by ecology. ISME J.

[46] Gueimonde M, Sánchez B (2013). G de Los Reyes-Gavilán C. Margolles A Antibiotic resistance in probiotic bacteria Front Microbiol.

[47] Martinez JL, Baquero F (2000). Mutation frequencies and antibiotic resistance. Antimicrob Agents Chemother.

[48] Cotter Paul D., Hill Colin, Ross R. Paul (2006). What's in a name? Class distinction for bacteriocins. Nature Reviews Microbiology.

[49] Heng Nicholas C. K., Tagg John R. (2006). What's in a name? Class distinction for bacteriocins. Nature Reviews Microbiology.

[50] Field D, Cotter PD, Hill C, Ross RP (2015). Bioengineering Lantibiotics for therapeutic success. Front Microbiol.

[51] McAuliffe O, Ryan MP, Ross RP, Hill C, Breeuwer P, Abee T (1998). Lacticin 3147, a broad-spectrum bacteriocin which selectively dissipates the membrane potential. Appl Environ Microbiol.

[52] Masuda Y, Ono H, Kitagawa H, Ito H, Mu F, Sawa N (2011). Identification and characterization of leucocyclicin Q, a novel cyclic bacteriocin produced by *Leuconostoc mesenteroides* TK41401. Appl Environ Microbiol.

[53] Shelburne CE, An FY, Dholpe V, Ramamoorthy A, Lopatin DE, Lantz MS (2006). The spectrum of antimicrobial activity of the bacteriocin subtilosin a. J Antimicrob Chemother.

[54] Liu Z, Budiharjo A, Wang P, Shi H, Fang J, Borriss R (2013). The highly modified microcin peptide plantazolicin is associated with nematicidal activity of *Bacillus amyloliquefaciens* FZB42. Appl Microbiol Biotechnol.

[55] Kawai Y, Kemperman R, Kok J, Saito T (2004). The circular bacteriocins gassericin a and circularin a. Curr Protein Pept Sci.

[56] Kemperman R, Kuipers A, Karsens H, Nauta A, Kuipers O, Kok J (2003). Identification and characterization of two novel clostridial bacteriocins, circularin a and closticin 574. Appl Environ Microbiol.

[57] Begley M, Cotter PD, Hill C, Ross RP (2009). Identification of a novel two-peptide lantibiotic, lichenicidin, following rational genome mining for LanM proteins. Appl Environ Microbiol.

[58] Simmonds RS, Pearson L, Kennedy RC, Tagg JR (1996). Mode of action of a lysostaphin-like bacteriolytic agent produced by *Streptococcus zooepidemicus* 4881. Appl Environ Microbiol.

[59] Liu W-T, Yang Y-L, Xu Y, Lamsa A, Haste NM, Yang JY (2010). Imaging mass spectrometry of intraspecies metabolic exchange revealed the cannibalistic factors of *Bacillus subtilis*. Proc Natl Acad Sci U S A.

[60] González-Pastor JE, Hobbs EC, Losick R (2003). Cannibalism by sporulating bacteria. Science.

[61] Lin D, Qu LJ, Gu H, Chen Z (2001). A 3.1-kb genomic fragment of *Bacillus subtilis* encodes the protein inhibiting growth of Xanthomonas oryzae pv. Oryzae. J Appl Microbiol.

[62] Rossi M, Amaretti A, Raimondi S (2011). Folate production by probiotic Bacteria. Nutrients.

[63] Zhu T, Pan Z, Domagalski N, Koepsel R, Ataai MM, Domach MM (2005). Engineering of *Bacillus subtilis* for enhanced total synthesis of folic acid. Appl Environ Microbiol.

[64] Ogata H, Goto S, Sato K, Fujibuchi W, Bono H, Kanehisa M (1999). KEGG: Kyoto encyclopedia of genes and genomes. Nucleic Acids Res.

[65] de Crécy-Lagard V (2014). Variations in metabolic pathways create challenges for automated metabolic reconstructions: examples from the tetrahydrofolate synthesis pathway. Comput Struct Biotechnol J.

[66] Salem AR, Pattison JR, Foster MA (1972). Folic acid and the methylation of homocysteine by *Bacillus subtilis*. Biochem J.

[67] Khatri I, Sharma S, Ramya TNC, Subramanian S (2016). Complete genomes of *Bacillus coagulans* S-lac and *Bacillus subtilis* TO-A JPC, Two Phylogenetically Distinct Probiotics. PLoS One.

[68] Casanovas-Massana A, Sala-Comorera L, Blanch AR (2014). Quantification of tetracycline and chloramphenicol resistance in digestive tracts of bulls and piglets fed with Toyocerin®, a feed additive containing *Bacillus toyonensis* spores. Vet Microbiol.

[69] Zhang S, Hu Y, Fan Q, Wang X, He J (2015). Two-component system YvqEC-dependent bacterial resistance against vancomycin in *Bacillus thuringiensis*. Antonie Van Leeuwenhoek.

[70] Abriouel H, Franz CMAP (2011). Omar N ben, Gálvez a. diversity and applications of *Bacillus* bacteriocins. FEMS Microbiol Rev.

[71] Chin CS, Alexander DH, Marks P, Klammer AA, Drake J, Heiner C (2013). Nonhybrid, finished microbial genome assemblies from long-read SMRT sequencing data. Nat Methods.

[72] Urdaci MC, Bressollier P, Pinchuk I (2004). *Bacillus clausii* Probiotic Strains. J Clin Gastroenterol.

[73] Ghelardi E, Celandroni F, Salvetti S, Gueye SA, Lupetti A, Senesi S (2015). Survival and persistence of *Bacillus clausii* in the human gastrointestinal tract following oral administration as spore-based probiotic formulation. J Appl Microbiol.

[74] Vecchione A, Celandroni F, Mazzantini D, Senesi S, Lupetti A, Ghelardi E (2018). Compositional quality and potential gastrointestinal behavior of probiotic products commercialized in Italy. Front Med.

[75] Gargis AS, O’Rourke A-LD, Sloan GL, Simmonds RS (2009). Prevalence and acquisition of the genes for zoocin a and zoocin a resistance in *Streptococcus equi* subsp. *zooepidemicus*. J Mol Evol.

[76] Williams LD, Burdock GA, Jiménez G, Castillo M (2009). Literature review on the safety of Toyocerin®, a non-toxigenic and non-pathogenic Bacillus cereus var. *toyoi* preparation. Regul Toxicol Pharmacol.

[77] Fuchs CS, Willett WC, Colditz GA, Hunter DJ, Stampfer MJ, Speizer FE (2002). The influence of folate and multivitamin use on the familial risk of colon cancer in women. Cancer Epidemiol Biomark Prev.

[78] White E, Shannon JS, Patterson RE, Hunter DJ, Stampfer MJ, Speizer FE (1997). Relationship between vitamin and calcium supplement use and colon cancer. Cancer Epidemiol Biomark Prev.

[79] Schallmey M, Singh A, Ward OP (2004). Developments in the use of *Bacillus* species for industrial production. Can J Microbiol.

[80] Kellner R, JUNG G, HORNER T, ZAHNER H, SCHNELL N, K-D ENTIAN (1988). Gallidermin: a new lanthionine-containing polypeptide antibiotic. Eur J Biochem.

[81] Kalyon B, Helaly SE, Scholz R, Nachtigall J, Vater J, Borriss R (2011). Plantazolicin a and B: structure elucidation of Ribosomally synthesized Thiazole/Oxazole peptides from *Bacillus amyloliquefaciens* FZB42. Org Lett.

[82] Alvarez-Ordóñez A, Begley M, Clifford T, Deasy T, Considine K, O’Connor P (2014). Investigation of the antimicrobial activity of *Bacillus licheniformis* strains isolated from retail powdered infant Milk formulae. Probiotics Antimicrob Proteins.

[83] Aziz RK, Bartels D, Best AA, DeJongh M, Disz T, Edwards RA (2008). The RAST server: rapid annotations using subsystems technology. BMC Genomics.

[84] Overbeek R, Olson R, Pusch GD, Olsen GJ, Davis JJ, Disz T (2014). The SEED and the rapid annotation of microbial genomes using subsystems technology (RAST). Nucleic Acids Res.

[85] Lowe TM, Eddy SR (1997). tRNAscan-SE: a program for improved detection of transfer RNA genes in genomic sequence. Nucleic Acids Res.

[86] Lagesen K, Hallin P, Rodland EA, Staerfeldt HH, Rognes T, Ussery DW (2007). RNAmmer: consistent and rapid annotation of ribosomal RNA genes. Nucleic Acids Res.

[87] Altschul SF, Gish W, Miller W, Myers EW, Lipman DJ, Yu Y (1990). Basic local alignment search tool. J Mol Biol.

[88] Antipov D, Hartwick N, Shen M, Raiko M, Lapidus A, Pevzner PA (2016). plasmidSPAdes: assembling plasmids from whole genome sequencing data. Bioinformatics.

[89] Edgar RC (2004). MUSCLE: multiple sequence alignment with high accuracy and high throughput. Nucleic Acids Res.

[90] Stamatakis A (2014). RAxML version 8: a tool for phylogenetic analysis and post-analysis of large phylogenies. Bioinformatics..

[91] Tatusov RL, Fedorova ND, Jackson JD, Jacobs AR, Kiryutin B, Koonin EV (2003). The COG database: an updated version includes eukaryotes. BMC Bioinformatics.

[92] Eddy SR (1996). Hidden Markov models. Curr Opin Struct Biol.

[93] Eddy SR (2011). Accelerated profile HMM searches. PLoS Comput Biol.

[94] McArthur AG, Waglechner N, Nizam F, Yan A, Azad MA, Baylay AJ (2013). The comprehensive antibiotic resistance database. Antimicrob Agents Chemother.

[95] van Heel AJ, de Jong A, Montalbán-López M, Kok J, Kuipers OP (2013). BAGEL3: Automated identification of genes encoding bacteriocins and (non-)bactericidal posttranslationally modified peptides. Nucleic Acids Res.

[96] Kanehisa M, Goto S, Kawashima S, Okuno Y, Hattori M (2004). The KEGG resource for deciphering the genome. Nucleic Acids Res.

