# Supplementary material for: Composite genome sequence of Bacillus clausii, a probiotic commercially available as Enterogermina®, and insights into its probiotic properties
Source: BMC Microbiol. 2019 Dec 30;19:307. doi: 10.1186/s12866-019-1680-7 (PMC6937992; doi:10.1186/s12866-019-1680-7)
Supplement: Supplementary file 1 — Additional file 1: Figure S1A. Mauve alignment of B. clausii ENTPro with B. clausii B106 Figure S1B. Mauve alignment of B. clausii ENTPro with B. clausii KSM K-16. Figure S2. The 16S rRNA alignment of operons identified in B. clausii ENTPro and its parental strains. The V-regions are identified using V-Xtractor v. 2.1 67. The V regions are marked by red bars and mutated positions are marked in pink color. Figure S3. RpoB sequence alignment between E. coli and B. clausii genomes. Figure S4. The Streptomycin biosynthesis pathway components present in B. clausii. Green box marks the presence of enzymes present in B. clausii. Figure S5. The Vancomycin resistance pathway components present in B. toyonensis BCT-7112. Green box marks the presence of gene whereas the not filled box marks the absence. Table S1. Genome Similarity of B. clausii ENTPro with other B. clausii genomes. Formula I, II and III represents different methods used by GGDC to calculate the similarities. Table S2. Distribution of proteins involved in probiotic properties in B. clausii ENTPro genome. Table S3. Proteins and domains involved in conferring antibiotic resistance in B. clausii ENTPro. Table S4. Accession numbers of the genomes used to generate the phylogenies. [file 12866_2019_1680_MOESM1_ESM.pdf]

BMC Microbiology

Supplementary Information

Composite genome sequence of *Bacillus clausii*, a probiotic commercially available as Enterogermina®, and insights into its probiotic properties

Indu Khatri[1,2*], Gaurav Sharma[1,3*], Srikrishna Subramanian[1#]

* Equal Contribution
[#] Corresponding author

Affiliations:
[1] CSIR-Institute of Microbial Technology, Sector-39A, Chandigarh, India
[2] Leiden University Medical Center, Leiden, the Netherlands
[3] Institute of Bioinformatics and Applied Biotechnology, Bengaluru, Karnataka, India

Contact Information:

Srikrishna Subramanian
Institute of Microbial Technology-CSIR
Sector-39A, Chandigarh
India-160036
Email: krishna@imtech.res.in
Telephone: +911726665483

Figure S1: Mauve alignment of *B. clausii* ENTPro with its closer genomes. A) *B.clausii* ENTPro with *B. clausii* B106 **B**) *B.clausii* ENTPro with *B. clausii* KSM K-16.

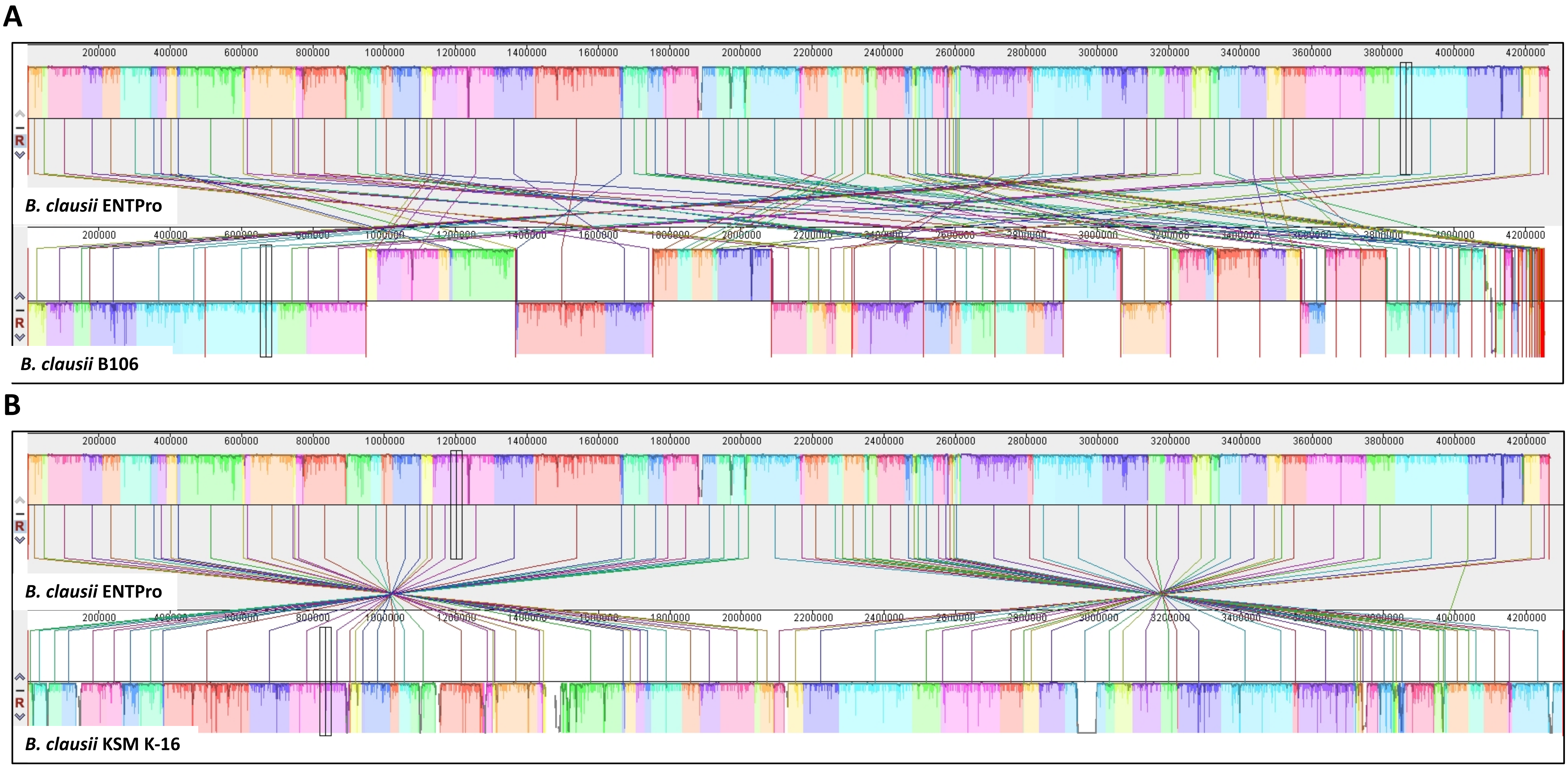

Figure S2: The 16S rRNA alignment of operons identified in *B. clausii* ENTPro and its parental strains. The V-region are identified using V-Xtractor v.2.1 67. The V regions are marked by red bars and mutated positions are marked in pink color.

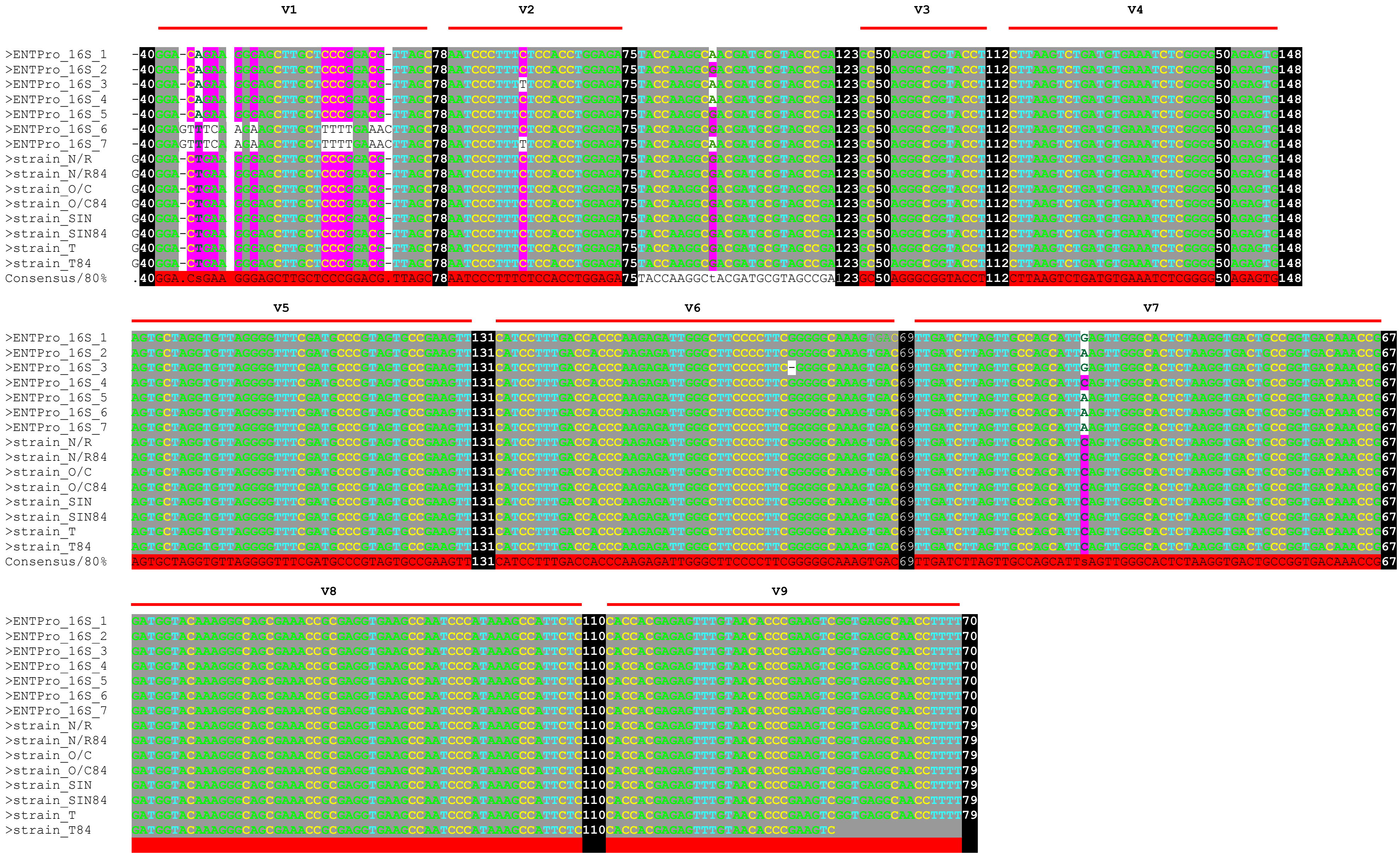

Figure S3: RpoB sequence alignment between *E. coli* and *B. clausii* genomes.

-------RIF resistance determining region (RRDR)-------

With reference to *Escherichia coli* str. K-12

Protein Id	Species	--------------Cluster I--------------- (507–533)	--Cluster II-- (563, 567, 572)	-Cluster III- (687)
YP_491474.1	*Escherichia coli str. K-12*	GSSQLSQFMDQNNPLSEITHKRRISAL	TPEGPNIGLI	R
ALA52738.1	*B. clausii ENTPro*	GSSQLSQFMDQTNPLAELTHKRRLSAL	TPEGLNIGLI	R
BAD62685.1	*B. clausii KSM-K16*	GSSQLSQFMDQTNPLAELTHKRRLSAL	TPEGPNIGLI	R
WP_026314572.1	*B. acidiproducens DSM 23148*	GSSQLSQFMDQTNPLAELTHKRRLSAL	TPEGPNIGLI	R
WP_026678032.1	*B. gelatini 15865*	GSSQLSQFMDQTNPLAELTHKRRLSAL	TPEGPNIGLI	R
WP_026702406.1	*B. aidingensis DSM 18341*	GSSQLSQFMDQTNPLAELTHKRRLSAL	TPEGPNIGLI	R
WP_035664720.1	*B. akibai JCM 9157*	GSSQLSQFMDQTNPLAELTHKRRLSAL	TPEGPNIGLI	R
WP_003322601.1	*B. alcalophilus ATCC 27647*	GSSQLSQFMDQTNPLAELTHKRRLSAL	TPEGPNIGLI	R
WP_113767304.1	*B. altitudinis 41KF2b*	GSSQLSQFMDQTNPLAELTHKRRLSAL	TPEGPNIGLI	R
WP_071348597.1	*B. amyloliquefaciens B1895*	GSSQLSQFMDQTNPLAELTHKRRLSAL	TPEGPNIGLI	R
WP_000147548.1	*B. anthracis 2000031021*	GSSQLSQFMDQTNPLAELTHKRRLSAL	TPEGPNIGLI	R
WP_003328296.1	*B. atrophaeus 1013-1*	GSSQLSQFMDQTNPLAELTHKRRLSAL	TPEGPNIGLI	R
WP_026688633.1	*B. aurantiacus DSM 18675*	GSSQLSQFMDQTNPLAELTHKRRLSAL	TPEGPNIGLI	R
WP_039957468.1	*B. azotoformans LMG 9581*	GSSQLSQFMDQTNPLAELTHKRRLSAL	TPEGPNIGLI	R
WP_035198181.1	*B. azotoformans MEV2011*	GSSQLSQFMDQTNPLAELTHKRRLSAL	TPEGPNIGLI	R
WP_007085298.1	*B. bataviensis LMG 21833*	GSSQLSQFMDQTNPLAELTHKRRLSAL	TPEGPNIGLI	R
WP_026673488.1	*B. bogoriensis ATCC BAA-922*	GSSQLSQFMDQTNPLAELTHKRRLSAL	TPEGPNIGLI	R
WP_023626439.1	*B. boroniphilus JCM 21738*	GSSQLSQFMDQTNPLAELTHKRRLSAL	TPEGPNIGLI	R
WP_013486742.1	*B. cellulosilyticus DSM 2522*	GSSQLSQFMDQTNPLAELTHKRRLSAL	TPEGPNIGLI	R
AJG54130.1	*B. cereus 03BB102*	GSSQLSQFMDQTNPLAELTHKRRLSAL	TPEGPNIGLI	R
WP_026696947.1	*B. chagannorensis DSM 18086*	GSSQLSQFMDQTNPLAELTHKRRLSAL	TPEGPNIGLI	R
WP_029282847.1	*B. cibi DSM 16189*	GSSQLSQFMDQTNPLAELTHKRRLSAL	TPEGPNIGLI	R
WP_017551225.1	*B. coagulans 2-6*	GSSQLSQFMDQTNPLAELTHKRRLSAL	TPEGPNIGLI	R
WP_010170030.1	*B. coahuilensis m4-4*	GSSQLSQFMDQTNPLAELTHKRRLSAL	TPEGPNIGLI	R
WP_048721547.1	*B. cytotoxicus NVH 391-98*	GSSQLSQFMDQTNPLAELTHKRRLSAL	TPEGPNIGLI	R
WP_019394077.1	*B. endophyticus 2102*	GSSQLSQFMDQTNPLGELTHKRRLSAL	TPEGPNIGLI	R
WP_035332308.1	*B. firmus DS1*	GSSQLSQFMDQTNPLAELTHKRRLSAL	TPEGPNIGLI	R
WP_119542719.1	*B. flexus 27Col1.1E*	GSSQLSQFMDQTNPLGELTHKRRLSAL	TPEGPNIGLI	R
WP_018708719.1	*B. fordii DSM 16014*	GSSQLSQFMDQTNPLAELTHKRRLSAL	TPEGPNIGLI	R
WP_033677188.1	*B. gaemokensis*	GSSQLSQFMDQTNPLAELTHKRRLSAL	TPEGPNIGLI	R
WP_025729854.1	*B. ginsengihumi J57*	GSSQLSQFMDQTNPLAELTHKRRLSAL	TPEGPNIGLI	R
WP_010896309.1	*B. halodurans C-125*	GSSQLSQFMDQTNPLAELTHKRRLSAL	TPEGPNIGLI	R
GAE31479.1	*B. hemicellulosilyticus JCM 915*	GSSQLSQFMDQTNPLAELTHKRRLSAL	TPEGPNIGLI	R
WP_029282847.1	*B. indicus LMG 22858*	GSSQLSQFMDQTNPLAELTHKRRLSAL	TPEGPNIGLI	R
AGX02158.1	*B. infantis NRRL B-14911*	GSSQLSQFMDQTNPLAELTHKRRLSAL	TPEGPNIGLI	R
WP_008406991.1	*B. isronensis B3W22*	GSSQLSQFMDQTNPLAELTHKRRLSAL	TPEGPNIGLI	R
WP_035322261.1	*B. kribbensis DSM 17871*	GSSQLSQFMDQTNPLAELTHKRRLSAL	TPEGPNIGLI	R
AIC92646.1	*B. lehensis G1*	GSSQLSQFMDQTNPLAELTHKRRLSAL	TPEGPNIGLI	R
WP_011197480.1	*B. licheniformis 10-1-A*	GSSQLSQFMDQTNPLAELTHKRRLSAL	TPEGPNIGLI	R
WP_007203559.1	*B. macauensis ZFHKF-1*	GSSQLSQFMDQTNPLAELTHKRRLSAL	TPEGPNIGLI	R
WP_034642626.1	*B. manliponensis JCM 15802*	GSSQLSQFMDQTNPLAELTHKRRLSAL	TPEGPNIGLI	R
WP_025028113.1	*B. mannanilyticus JCM 10596*	GSSQLSQFMDQTNPLAELTHKRRLSAL	TPEGPNIGLI	R
WP_022630048.1	*B. marmarensis DSM 21297*	GSSQLSQFMDQTNPLAELTHKRRLSAL	TPEGPNIGLI	R
WP_019244369.1	*B. massilioanorexius AP8*	GSSQLSQFMDQTNPLAELTHKRRLSAL	TPEGPNIGLI	R
WP_019157078.1	*B. massiliosenegalensis JC6*	GSSQLSQFMDQTNPLAELTHKRRLSAL	TPEGPNIGLI	R
KFM94933.1	*B. megaterium BMS*	GSSQLSQFMDQTNPLGELTHKRRLSAL	TPEGPNIGLI	R
WP_003348636.1	*B. methanolicus MGA3*	GSSQLSQFMDQTNPLAELTHKRRLSAL	TPEGPNIGLI	R
AIE58634.1	*B. methylotrophicus SK19.001*	GSSQLSQFMDQTNPLAELTHKRRLSAL	TPEGPNIGLI	R
WP_010332764.1	*B. mojavensis RO-H-1*	GSSQLSQFMDQTNPLAELTHKRRLSAL	TPEGPNIGLI	R
WP_144505032.1	*B. mycoides 219298*	GSSQLSQFMDQTNPLAELTHKRRLSAL	TPEGPNIGLI	R
WP_016204550.1	*B. nealsonii AAU1*	GSSQLSQFMDQTNPLAELTNKRRLSAL	TPEGPNIGLI	R
AND37748.1	*B. oceanisediminis 2691*	GSSQLSQFMDQTNPLAELTHKRRLSAL	TPEGPNIGLI	R
WP_028400850.1	*B. panaciterrae DSM 19096*	GSSQLSQFMDQTNPLAELTHKRRLSAL	TPEGPNIGLI	R
WP_012957129.1	*B. pseudofirmus OF4*	GSSQLSQFMDQTNPLAELTHKRRLSAL	TPEGPNIGLI	R
EEM18962.1	*B. pseudomycoides DSM 12442*	GSSQLSQFMDQTNPLAELTHKRRLSAL	TPEGPNIGLI	R
WP_034666728.1	*B. pumilus 3-19*	GSSQLSQFMDQTNPLAELTHKRRLSAL	TPEGPNIGLI	R
KEP31695	*B. safensis CFA06*	GSSQLSQFMDQTNPLAELTHKRRLSAL	TPEGPNIGLI	R
WP_013171085.1	*B. selenitireducens MLS10*	GSSQLSQFMDQTNPLAELTHKRRLSAL	TPEGPNIGLI	R
WP_003156440.1	*B. siamensis KCTC 13613*	GSSQLSQFMDQTNPLAELTHKRRLSAL	TPEGPNIGLI	R
WP_003354794.1	*B. smithii 7 3 47FAA*	GSSQLSQFMDQTNPLAELTHKRRLSAL	TPEGPNIGLI	R
WP_006640310.1	*B. sonorensis L12*	GSSQLSQFMDQTNPLAELTHKRRLSAL	TPEGPNIGLI	R
EMI15076.1	*B. stratosphericus LAMA 585*	GSSQLSQFMDQTNPLAELTHKRRLSAL	TPEGPNIGLI	R
WP_087997849.1	*B. subtilis B7-s*	GSSQLSQFMDQTNPLAELTHKRRLSAL	TPEGPNIGLI	R
WP_024714043.1	*B. tequilensis KCTC 13622*	GSSQLSQFMDQTNPLAELTHKRRLSAL	TPEGPNIGLI	R
ADH04916.1	*B. thuringiensis BMB171*	GSSQLSQFMDQTNPLAELTHKRRLSAL	TPEGPNIGLI	R
WP_010285026.1	*B. timonensis MM10403188*	GSSQLSQFMDQTNPLAELTHKRRLSAL	TPEGPNIGLI	R
AHA08759.1	*B. toyonensis BCT-7112*	GSSQLSQFMDQTNPLAELTHKRRLSAL	TPEGPNIGLI	R
GYS_RS21080	*B. vallismortis DV1-F-3*	GSSQLSQFMDQTNPLAELTHKRRLSAL	TPEGPNIGLI	R
WP_034766001.1	*B. vietnamensis HD-02*	GSSQLSQFMDQTNPLAELTHKRRLSAL	TPEGPNIGLI	R
WP_024027914.1	*B. vireti LMG 21834*	GSSQLSQFMDQTNPLAELTHKRRLSAL	TPEGPNIGLI	R
WP_034745200.1	*B. wakoensis JCM 9140*	GSSQLSQFMDQTNPLAELTHKRRLSAL	TPEGPNIGLI	R
ETT84540.1	*B. weihenstephanensis FSL H7-68*	GSSQLSQFMDQTNPLAELTHKRRLSAL	TPEGPNIGLI	R
Protein Id	Consensus/80%	GSSQLSQFMDQTNPLAELTHKRRLSAL	TPEGPNIGLI	R

Figure S4: The Streptomycin synthesis pathway components present in *B. clausii.* Green box marks the presence of enzymes present in *B. clausii*.

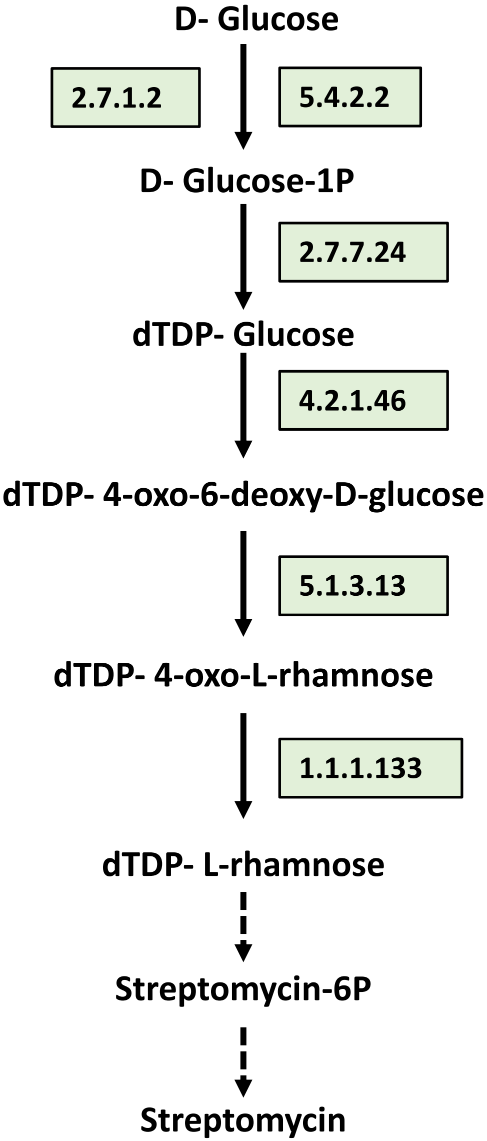

Figure S5: The Vancomycin resistance pathway components present in *B. toyonensis* BCT-7112. Green box marks the presence of gene whereas the not filled box marks the absence.

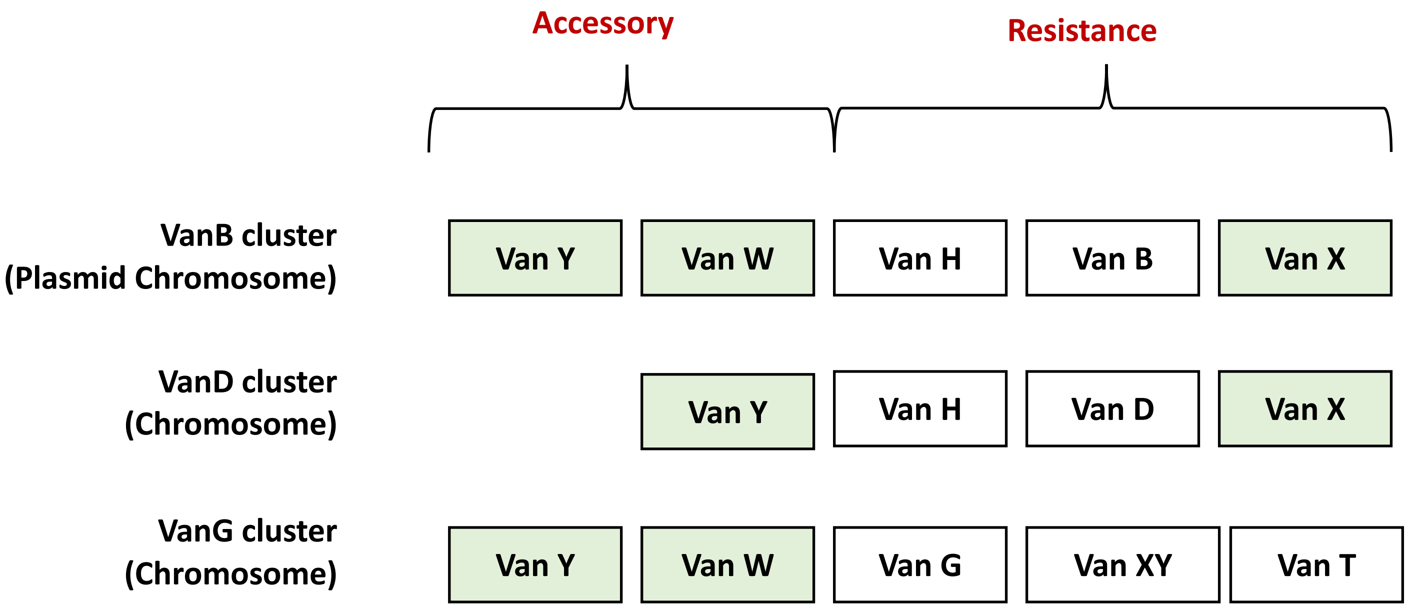

Table S1: Genome Similarity of *B. clausii* ENTPro with other *B. clausii* genomes. Formula I, II and III represents different methods used by GGDC to calculate the similarities.

Query Genome (GCA_000737305.2)	Reference Genome	Assembly reference	Formula I	Formula II	Formula III
B. clausii ENTPro	*B. clausii* 7520-2	GCA_002272085.1	85.6	62.4	84
B. clausii ENTPro	*B. clausii* 7522	GCA_002272655.1	87.4	63.1	85.6
B. clausii ENTPro	*B. clausii* 7523-2	GCA_002272645.1	76.9	62.8	76.7
B. clausii ENTPro	*B. clausii* 7529	GCA_002272615.1	92.7	92.8	94.9
B. clausii ENTPro	*B. clausii* 7535-K	GCA_002272605.1	85.8	62.7	84.2
B. clausii ENTPro	*B. clausii* 7537-T	GCA_002272525.1	89.6	92.1	92.6
B. clausii ENTPro	*B. clausii* 7538	GCA_002272545.1	93.3	93.5	95.4
B. clausii ENTPro	*B. clausii* 7539	GCA_002272535.1	86.4	62.7	84.7
B. clausii ENTPro	*B. clausii* 7540-1	GCA_002271585.1	87	63.4	85.3
B. clausii ENTPro	*B. clausii* 7540-2	GCA_002271575.1	95.5	93.1	96.9
B. clausii ENTPro	*B. clausii* 7541	GCA_002271555.1	94.5	92.4	96.1
B. clausii ENTPro	*B. clausii* 7543	GCA_002272505.1	86.9	63.4	85.3
B. clausii ENTPro	*B. clausii* 7547-G	GCA_002272445.1	93.5	93.4	95.5
B. clausii ENTPro	*B. clausii* 7894-1	GCA_002271695.1	86.7	63.4	85.1
B. clausii ENTPro	*B. clausii* AKU0647	GCA_001598215.1	75.9	50.7	72.4
B. clausii ENTPro	*B. clausii* B106	GCA_002266625.1	99.9	99.8	100
B. clausii ENTPro	*B. clausii* DSM-8716	GCA_002250115.1	86.2	62.6	84.5
B. clausii ENTPro	*B. clausii* KSM-K16	GCA_000009825.1	95.2	94.3	96.8
B. clausii ENTPro	*B. clausii* UBBC-07	GCA_000981315.1	93.1	92	95.1

Table S2: Distribution of proteins involved in probiotic properties in *B. clausii* ENTPro genome.

	Probiotic nature	Accession Numbers	Domain-tag	Domains-identified	Domain-wise E value (hmmscan)
Environmental stress resistance	Chaperonins GroEL	ALA51946	PF00118	Cpn60_TCP1	4.30E-104
	Chaperonins GroES	ALA51947	PF00166	Cpn10	7.30E-37
	Clp protease	ALA52850	PF00574	CLP_protease	2.90E-79
		ALA54099	PF00574	CLP_protease	3.40E-89
		ALA54894	PF00574	CLP_protease	5.10E-36
	Cold shock protein	ALA52002	PF00313	CSD	1.10E-31
		ALA54053	PF00313	CSD	3.50E-33
	Heat shock protein	ALA52772	PF01430	HSP33	4.20E-90
	Heat resistance	ALA51177	PF01025	GrpE	6.20E-52
	Hyperosmotic stress	ALA51175	PF00226	DnaJ	4.40E-25
	Oxidative stress	ALA51081	PF01625	PMSR	4.20E-60
		ALA54975	PF01625	PMSR	2.50E-60
	Universal Stress	ALA52046	PF00582	Usp	1.20E-07
		ALA52818	PF00582	Usp	4.20E-06
		ALA53908	PF00582	Usp	3.20E-32
Adhesion to gut mucosa	Mucus binding protein	ALA51653	PF00746	Gram_pos_anchor	9.10E-05
	Collagen binding protein	ALA53512	PF05737	Collagen_bind	1.60E-30
	Fibronectin binding protein	ALA54776	PF05833	FbpA	1.10E-166
	Sortase	ALA51654	PF04203	Sortase	4.50E-24
		ALA51828	PF04203	Sortase	1.00E-26
		ALA53120	PF04203	Sortase	4.50E-28
		ALA53513	PF04203	Sortase	5.60E-26
		ALA53680	PF04203	Sortase	1.50E-22
	Flagellin	ALA53337	PF00669	Flagellin_N	1.90E-40
		ALA54045	PF00669	Flagellin_N	4.90E-16
	Triosephosphate isomerase	ALA54107	PF00121	TIM	3.00E-95

**Bile tolerance**	Ornithine decarboxylase	ALA52835	PF01276	OKR_DC_1	1.60E-60
		ALA54693	PF01276	OKR_DC_1	1.90E-101
	Sodium bile acid symporter family	ALA51223	PF01758	SBF	1.10E-14
		ALA51591	PF01758	SBF	1.40E-41
		ALA52219	PF01758	SBF	3.80E-25
**Acid tolerance**	F0F1 ATP_synthase	ALA53170	PF00119	ATP-synt_A	3.20E-49
		ALA53171	PF00137	ATP-synt_C	7.10E-14
		ALA53172	PF00430	ATP-synt_B	3.10E-40
		ALA53173	PF00213	OSCP	1.70E-49
		ALA53174	PF00006	ATP-synt_ab	2.10E-73
		ALA53175	PF00231	ATP-synt	1.20E-91
		ALA53176	PF00006	ATP-synt_ab	1.70E-68
		ALA53177	PF02823	ATP-synt_DE_N	1.40E-25

Table S3: Proteins and domains involved in conferring antibiotic resistance in *B. clausii* ENTPro.

Resistance to	Accession Numbers	Domain-tag	Domains-identified	Domain-wise E value (hmmscan)	Homolog in KSM-K16 (% identity)
Aminoglycosidase	ALA51464	PF01636	APH	8.1E-19	ABC_RS07395 (97.31)
Aminoglycosidase	ALA51859	PF01636	APH	9.9E-25	ABC_RS05095 (100)
Aminoglycosidase	ALA51976	PF04439	Adenyl_transf	1.8E-117	ABC_RS04555 (99.65)
Aminoglycosidase	ALA52626	PF07827	KNTase_C	2E-62	ABC_RS01520 (96.09)
Penicillin represser	ALA52627	PF03965	Penicillinase_R	1.2E-34	ABC_RS01515 (98.41)
Penicillin regulatory	ALA52628	PF05569	Peptidase_M56	6.2E-57	ABC_RS01510 (94.35)
Penicillin	ALA52629	PF13354	Beta-lactamase2	8.4E-34	ABC_RS01505 (91.69)
Aminoglycosidase	ALA52671	PF01636	APH	6.6E-22	ABC_RS01175 (98.63)
Rifampicin	ALA52738	PF00562	RNA_pol_Rpb2_6	5E-167	ABC_RS00825 (100)
Tetracycline	ALA53309	cd00881	TetM_like	1.07E-131	ABC_RS19270 (98.92)
Aminoglycosidase	ALA53421	PF04439	Adenyl_transf	1.7E-124	ABC_RS18770 (97.23)
Aminoglycosidase	ALA53555	PF01636	APH	9.5E-21	ABC_RS18315 (97.8)
Erythromycin	ALA53582	PF00398	RrnaAD	1.7E-51	ABC_RS18170 (99.64)
Aminoglycosidase	ALA54163	PF01636	APH	2.5E-27	ABC_RS15455 (99.65)
Aminoglycosidase	ALA54297	PF01636	APH	2.1E-19	ABC_RS14455 (99.62)
Chloramphenicol	ALA55212	PF00302	CAT	3E-78	ABC_RS10335 (98.6)

Table S4: Accession numbers of the genomes used to generate the phylogenies.

Genome	GenBank assembly accession
B. altitudinis SGAir0031	GCA_002443015.2
B. amyloliquefaciens B1895	GCA_000696285.1
B. amyloliquefaciens H57	GCA_001617995.1
Bamyloliquefaciens-DSM7	GCA_000196735.1
B. anthracis str. Ames	GCA_000007845.1
B. anthracis str. Sterne	GCA_000008165.1
B. atrophaeus SRCM101359	GCA_002173495.1
B. beveridgei MLTeJB	GCA_001721685.1
B. bombysepticus str.Wang	GCA_000831065.1
B. cellulosilyticus DSM2522	GCA_000177235.2
B. cereus ATCC 14579	GCA_000007825.1
B. coagulans GB-I30	GCA_000756285.1
B. coagulans HM-08	GCA_000876545.1
B. coagulans S-lac	GCA_001039495.1
B. coagulans Unique IS-2	GCA_001578455.1
B. coagulans DSM1	GCA_000832905.1
B. cohnii DSM-6307	GCA_001591425.1
B. cytotoxicus NVH 391-98	GCA_000017425.1
B. endophyticus (filamentous) Hbe603	GCA_000972245.3
B. flexus KLBMP 4941	GCA_002024265.1
B. gibsonii FJAT-10019	GCA_001719185.1
B. glycinifermentans BGLY	GCA_900093775.1
B. gobiensis FJAT-4402	GCA_001278705.1
B. halodurans C-125	GCA_000011145.1
B. horikoshii 20a	GCA_002157855.1
B. infantis NRRL-B-14911	GCA_000473245.1
B. kochii BDGP4	GCA_002276165.1
B. krulwichiae AM31D	GCA_002109385.1
B. lehensis G1	GCA_000706725.1
B. licheniformis ATCC 14580	GCA_000011645.1
B. megaterium NBRC 15308	GCA_000832985.1
B. methanolicus MGA3	GCA_000724485.1
B. muralis G25-68	GCA_001645685.2
B. mycoides ATCC 6462	GCA_000832605.1
B. oceanisediminis 2691	GCA_000294775.2

B. paralicheniformis BL09	GCA_000876525.1
B. paralicheniformis 14DA11	GCA_002393225.1
B. paralicheniformis ATCC 9945a	GCA_000408885.1
B. pseudofirmus OF4	GCA_000005825.2
B. pseudomycoides DSM 12442	GCA_000161455.1
B. pumilus RI06-95	GCA_001183525.1
B. pumilus SH-B9	GCA_001578205.1
B. safensis KCTC 12796BP	GCA_001895885.1
B. simplex SH-B26	GCA_001578185.1
B. smithii DSM4216	GCA_001050115.1
B. sonorensis SRCM101395	GCA_002202015.1
B. subtilis CU1	GCA_002093785.1
B. subtilis KATMIRA1933	GCA_000691185.1
B. subtilis TO-A-JPC	GCA_001037985.1
B. subtilis 168	GCA_000009045.1
B. thuringiensis YBT-1518	GCA_000497525.2
B. thuringiensis serovar konkukian str.97-27	GCA_000008505.1
B. toyonensis BCT-7112	GCA_000496285.1
B. vallismortis NBIF-001	GCA_002113805.1
B. velezensis AS43.3	GCA_000319475.1
B. velezensis SB1216	GCA_001634675.1
B. velezensis SRCM10072	GCA_002201935.1
B. weihaiensis Alg07	GCA_001889165.1
B. xiamenensis VV3	GCA_001857925.1
B. kaustophilus HTA426	GCA_000009785.1
L. innocua Clip11262	GCA_000195795.1
L. ivanovii WSLC3009	GCA_000565155.1
L. marthii FSL S4-120	GCA_000183865.1
L. monocytogenes EGD-e	GCA_000196035.1
L. seeligeri serovar 1/2b str. SLCC3954	GCA_000027145.1
L. welshimeri NCTC11857	GCA_900187315.1
B. clausii 7520-2	GCA_002272085.1
B. clausii 7522	GCA_002272655.1
B. clausii 7523-2	GCA_002272645.1
B. clausii 7529	GCA_002272615.1
B. clausii 7535-K	GCA_002272605.1
B. clausii 7537-T	GCA_002272525.1
B. clausii 7538	GCA_002272545.1
B. clausii 7539	GCA_002272535.1

B. clausii 7540-1	GCA_002271585.1
B. clausii 7540-2	GCA_002271575.1
B. clausii 7541	GCA_002271555.1
B. clausii 7543	GCA_002272505.1
B. clausii 7547-G	GCA_002272445.1
B. clausii 7894-1	GCA_002271695.1
B. clausii AKU0647	GCA_001598215.1
B. clausii B106	GCA_002266625.1
B. clausii DSM-8716	GCA_002250115.1
B. clausii KSM-K16	GCA_000009825.1
B. clausii UBBC-07	GCA_000981315.1
B. clausii ENTPro	GCA_000737305.2
